# The Alzheimer’s therapeutic Lecanemab attenuates Aβ pathology by inducing an amyloid-clearing program in microglia

**DOI:** 10.1038/s41593-025-02125-8

**Published:** 2025-11-24

**Authors:** Giulia Albertini, Magdalena Zielonka, Marie-Lynn Cuypers, An Snellinx, Ciana Xu, Suresh Poovathingal, Marta Wojno, Kristofer Davie, Veerle van Lieshout, Katleen Craessaerts, Leen Wolfs, Emanuela Pasciuto, Tom Jaspers, Katrien Horré, Lurgarde Serneels, Mark Fiers, Maarten Dewilde, Bart De Strooper

**Affiliations:** 1 Centre for Brain and Disease Research, Flanders Institute for Biotechnology (VIB), Leuven, Belgium; 2 Department of Neurosciences and Leuven Brain Institute, KU Leuven, Leuven, Belgium; 3 Laboratory for Therapeutic and Diagnostic Antibodies, Department of Pharmaceutical and Pharmacological Sciences, KU Leuven, Leuven, Belgium; 4 Single Cell Core, Flanders Institute for Biotechnology (VIB), Leuven, Belgium; 5 Center for Molecular Neurology, Flanders Institute for Biotechnology (VIB), Antwerp, Belgium; 6 Department of Biomedical Sciences, University of Antwerp, Antwerp, Belgium; 7 UK Dementia Research Institute, University College London, London, UK; 8 Department of Human Genetics, KU Leuven, Leuven, Belgium

**Keywords:** Microglia, Alzheimer's disease, Alzheimer's disease

## Abstract

Controversies over anti-amyloid immunotherapies underscore the need to elucidate their mechanisms of action. Here we demonstrate that Lecanemab, a leading anti-β-amyloid (Aβ) antibody, mediates amyloid clearance by activating microglial effector functions. Using a human microglia xenograft mouse model, we show that Lecanemab significantly reduces Aβ pathology and associated neuritic damage, while neither fragment crystallizable (Fc)-silenced Lecanemab nor microglia deficiency elicits this effect despite intact plaque binding. Single-cell RNA sequencing and spatial transcriptomic analyses reveal that Lecanemab induces a focused transcriptional program that enhances phagocytosis, lysosomal degradation, metabolic reprogramming, interferon *γ* genes and antigen presentation. Finally, we identify *SPP1*/osteopontin as a major factor induced by Lecanemab treatment and demonstrate its role in promoting Aβ clearance. These findings highlight that effective amyloid removal depends on the engagement of microglia through the Fc fragment, providing critical insights for optimizing anti-amyloid therapies in Alzheimer’s disease.

## Abstract (web summary)

Lecanemab, a leading therapy for Alzheimer’s disease, induces an Fc-mediated transcriptional program in human microglia, linked to osteopontin and enhanced plaque phagocytosis, suggesting alternative strategies to promote plaque clearance.

## Main

Lecanemab, an antibody engineered to target soluble amyloid β-amyloid (Aβ) protofibrils^1^, effectively removes amyloid plaques from the brains of Alzheimer’s disease (AD) patients, slowing cognitive decline by 27%^2^. Although originally developed against peptides containing the rare Arctic mutation causally linked to inherited AD^1^, Lecanemab also shows efficacy in sporadic AD cases. Nonetheless, the precise mechanism by which its binding to Aβ oligomers leads to more effective Aβ-plaque clearance compared to other Aβ-binding antibodies^3^ remains unclear.

One prevailing hypothesis suggests that plaque clearance is mediated by fragment crystallizable (Fc) γ receptor (FcγR) activation of microglia, triggering phagocytosis of Aβ^4–6^. However, direct experimental evidence linking microglia activity to the therapeutic efficacy of Lecanemab is lacking. For instance, while some studies report microglia accumulation around amyloid plaques after immunotherapy^5^, such clustering is also observed without the antibody treatment and does not necessarily result in Aβ-plaque removal. Moreover, FcγR activation can induce a pro-inflammatory response with the release of cytokines and other toxic mediators^7,8^, potentially mitigating the benefits of Aβ clearance. Alternative FcγR-independent mechanisms of plaque removal have also been widely proposed^9–12^. Consequently, the exact mechanism by which Lecanemab clears amyloid plaques remains unresolved.

To uncover the mechanisms driving antibody-induced amyloid clearance, we generated Lecanemab^13^ alongside a human immunoglobulin G1 (IgG1) variant designed to abolish Fc-mediated effector functions^14,15^, Lecanemab LALA-PG. We leveraged our advanced human microglia xenotransplantation model for AD^16,17^. Using *Rag2*^*tm1.1Flv*^; *Csf1*^*tm1(CSF1)Flv*^; *Il2rg*^*tm1.1Flv*^; *App*^*tm3.1Tcs*^; *Csf1R*^*em1Bdes*^ mice, named hereafter as *App*^*NL-G-F*^ *Csf1r*^*ΔFIRE/ΔFIRE*^ mice, which lack endogenous microglia^17,18^, enabled us to assess these clinically relevant human antibodies directly. Notably, we corroborated our observations in fully immune-competent mice treated with mouse analogs of Lecanemab. Our work probes a critical conundrum, that is, why do microglia—although strongly activated in the presence of amyloid plaques—fail to clear these deposits? We hypothesize that antibody engagement unveils latent, transformative mechanisms, endowing microglia with an unexpected capacity for plaque clearance. Our work suggests that antibody treatment reprograms microglial function, providing insights into protective roles of these cells while also opening new avenues to understand and ultimately harness these changes for therapeutic benefit.

## Results

### Lecanemab binds to amyloid plaques

**Fig. 1:**
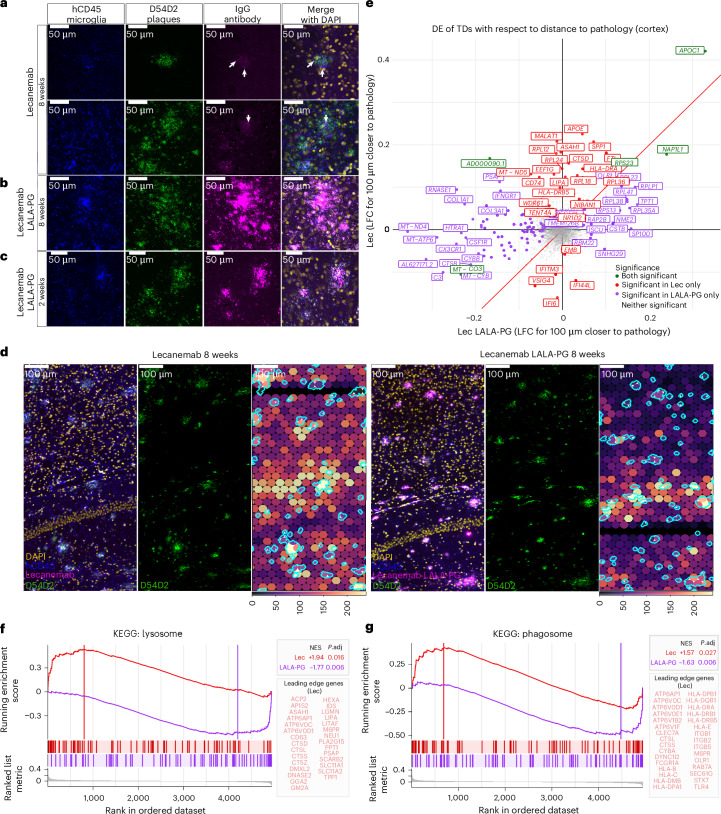
Lecanemab drives strong transcriptional changes in human microglia associated with Aβ plaques. **a**–**c**, Representative high-magnification confocal *z*-stacks of CD45 (human microglia, blue), D54D2 (Aβ, green), IgG (human antibody, magenta) and a merged view with DAPI-stained nuclei (yellow). These high-resolution *z*-stacks are used to show the colocalization between D54D2 and IgG, as well as the internalization of Lecanemab within the microglia. Scale bar = 50 µm. Staining shown is representative; experiments were performed on four mice per condition for 8-week treatments and three mice per condition for 2-week treatments, all from the same treatment batch, and the experiments were repeated across two independent staining batches. **a**, After 8 weeks of Lecanemab administration, human IgG is detected in the brain parenchyma, where it associates with Aβ plaques and is internalized by human microglia (arrows). **b**, Notably, after 8 weeks of Lecanemab LALA-PG administration, IgG exhibits strong accumulation on D54D2 + Aβ plaques, indicating that a functional Fc fragment is necessary for uptake in microglia. **c**, This accumulation is already apparent as early as 2 weeks after the treatment in Lecanemab LALA-PG-treated mice. **d**, Representative large-field images of the Nova-ST data coupled with immunofluorescence workflow for Lecanemab-treated (top) and Lecanemab LALA-PG-treated (bottom) mice. Immunofluorescence was performed to visualize human microglia (CD45^+^, blue), Aβ plaques (D54D2^+^, green), Lecanemab or Lecanemab LALA-PG (magenta) and DAPI-stained nuclei (yellow). D54D2 signal was used to define the plaque regions (outlined in cyan). Left, a merged view; middle, D54D2; right, spatial transcriptomic TDs (spots binned into hexbins with a diameter of 40 µm) overlayed with plaque ROIs. TDs are colored based on their relative expression of human transcripts (purple, low expression; yellow, high expression). Scale bar = 100 µm. Images shown are representative; experiments were performed on one mouse per condition in each of two independent Nova-ST batches (total two mice per condition). **e**, Quadrant plot showing the log_2_(FC) of genes in Lecanemab-treated (*x* axis) and Lecanemab LALA-PG-treated (*y* axis) TDs with respect to their distance to plaques in the Nova-ST dataset. TDs were analyzed in the cortical regions of *n* = 2 mice per condition. A positive log_2_(FC) indicates upregulation in proximity to plaques. Red, genes significant in Lecanemab microglia only; purple, genes significant in Lecanemab LALA-PG microglia only; green, genes significant in both comparisons; gray, genes not significant in either comparison. log_2_(FC) were calculated using edgeR’s quasi-likelihood *F* test (two-sided); *P* values were adjusted using the BH correction (*P*_adj_ < 0.05). **f**,**g**, From the differential gene expression analysis in **e**, we performed GSEA to further explore shifts in the microglia phenotype after the antibody treatment. We observed a significant positive enrichment of lysosome (**f**) and phagosome (**g**) genes in Lecanemab TDs near Aβ plaques (red), whereas such enrichment was not observed in Lecanemab LALA-PG TDs (purple). The vertical lines indicate the ES. Vertical tick marks along the *x* axis show the location of individual genes in the gene set within the log_2_(FC)-ranked gene list. The NES, two-sided *P*_adj_ value and the leading-edge genes are shown for the GSEA performed on the Lecanemab and Lecanemab LALA-PG TDs. *P* values were adjusted using the BH correction. FC, fold change; ES, enrichment score; NES, normalized enrichment score. BH, Benjamini–Hochberg. Source data

**Extended Data Fig. 1:**
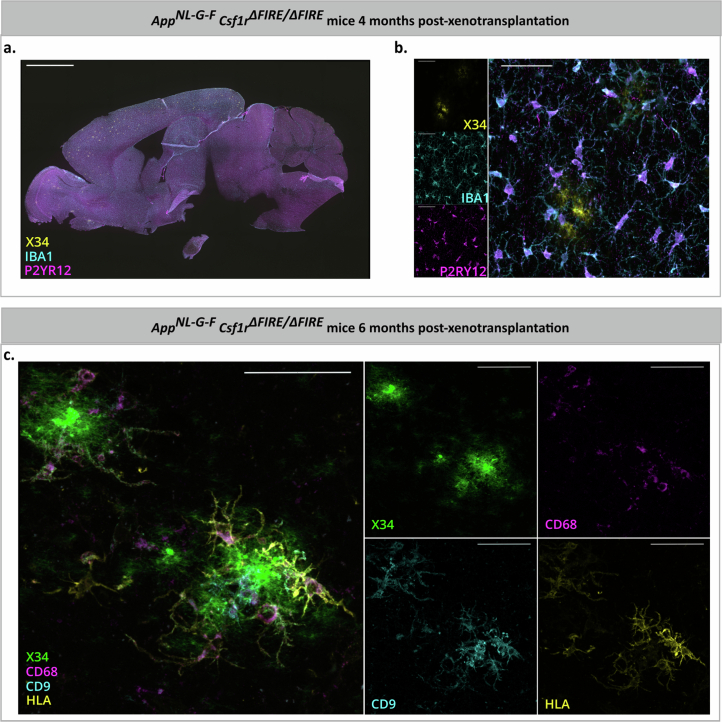
Human microglia in the brain of App^NL-G-F^ mice. **a**, Representative sagittal brain section showing the distribution of human engrafted microglia (labeled with human P2RY12, in magenta, and IBA1, in cyan) in App^NL-G-F^ Csf1r^ΔFIRE/ΔFIRE^ mice at 4 months of age (X-34^+^ plaques in yellow). Because these mice genetically lack mouse microglia, engraftment efficiency reaches 100%. Scale bar = 1 mm. **b**, Higher magnification confocal image of representative X-34 plaques (in yellow) surrounded by human microglia expressing IBA1 (cyan) and P2RY12 (magenta) at 4 months of age. Scale bar = 50 µm. **c**, Representative high-magnification confocal z-stacks of X-34 plaques (green) surrounded by human microglia expressing CD9 (cyan), HLA (yellow) and CD68 (magenta) at 6 months of age. Scale bar = 50 µm. Images shown are representative; sagittal sections at 4 months were obtained from 2 mice (**a**,**b**), high-magnification confocal z-stacks at 6 months from 4 mice (**c**).

By 4 months of age, xenografted human microglia had efficiently colonized the mouse brain (Extended Data Fig. 1a,b) and, by 6 months, they are fully able to mount amyloid responses that strongly resemble the ones observed in AD patients (as previously characterized in ref. ^16^; Extended Data Fig. 1c). Starting from 4 months of age, we administered weekly intraperitoneal injections of 10 mg kg^−1^ Lecanemab or Lecanemab LALA-PG^19^. After 8 weeks of treatment, we analyzed the distribution of the human antibodies in the brain parenchyma (Fig. 1a,b). Strikingly, while only sparse antibody signals were detected in Lecanemab-treated mice (notably within human CD45⁺ microglia surfaces, as reconstructed in Supplementary Video 1), Lecanemab LALA-PG strongly accumulated on Aβ plaques. This accumulation is already evident after 2 weeks of treatment (Fig. 1c). These data suggest that mutating the Fc fragment to abolish Fc-based effector functions prevents uptake of Lecanemab into microglia. Additionally, our findings demonstrate that Lecanemab binds to plaques, challenging the common assumption that it is specific to oligomers. These remarkable findings led us to investigate the potential changes these antibodies might induce in the human microglia surrounding the Aβ plaques^20,21^.

### Spatial transcriptomics reveals Lecanemab-driven enhancement of microglial phagocytic and lysosomal pathways

**Extended Data Fig. 2:**
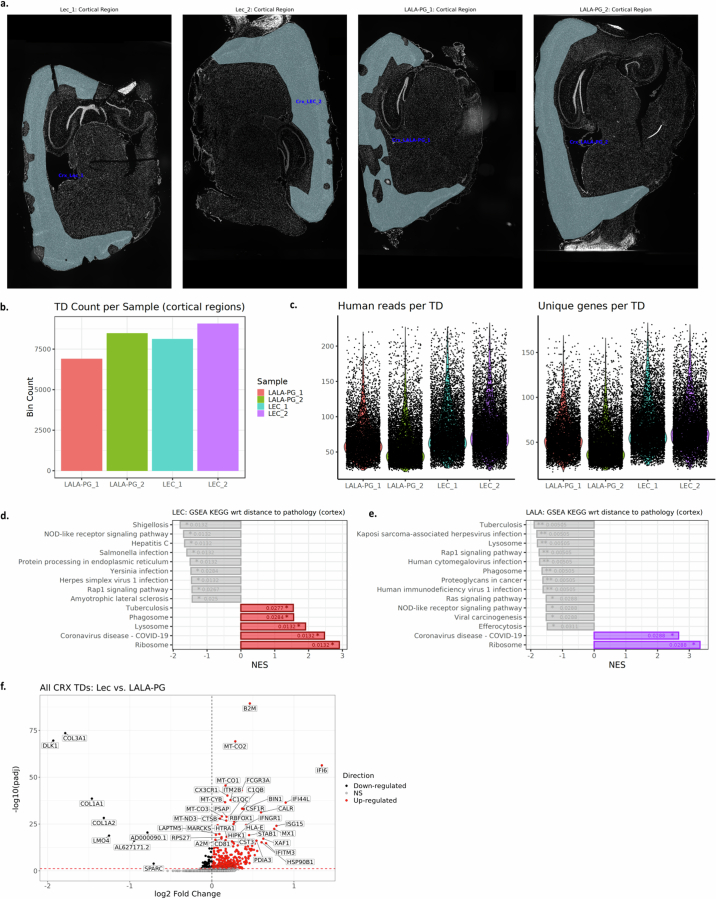
Supplementary information on Nova-ST experiment. **a**, Outline of the cortical regions (blue) selected for subsequent spatial transcriptomic analyses. Regions were manually delineated in QuPath to include cortical areas with high-quality stainings in all channels. **b**, Number of tissue domains (TDs, bin40s) per sample, where only TDs falling in specified cortical regions (**a**) and with at least 30 human transcripts were considered. **c**, Violin plots showing the distribution of the number of human UMIs and unique human genes in bin40s across the four samples. **d**,**e**, Normalized enrichment score (NES) of significantly (p.adj < 0.05) enriched KEGG pathways in function of cortical bins’ proximity to plaques in (**d**) Lecanemab and (**e**) Lecanemab LALA-PG-treated mice, as identified by gene set enrichment analysis (GSEA) on the differential gene expression results in Fig. 1e (*: p.adj < 0.05, **: p.adj < 0.01). Respective adjusted p-values for enrichments are specified. Two-sided p-values were adjusted using the Benjamini–Hochberg (BH) correction. **f**, Volcano plots showing a gene expression comparison between Lecanemab and Lecanemab LALA-PG TDs in cortical areas. Adjusted p-value threshold <0.05 (two-sided Wilcoxon rank-sum test, p-values adjusted with Bonferroni correction based on the total number of genes in the dataset). NS, not significant. Source data

We used Nova-ST^22^, a recently developed technique based on the Illumina NovaSeq flow cells, which enabled us to combine unbiased high-resolution spatial transcriptomics with immunofluorescence of amyloid plaques (positive for the anti-Aβ antibody D54D2) on the same tissue section (Fig. 1d and Extended Data Fig. 2). Because only the xenotransplanted cells are of human origin, all human-derived reads can be exclusively attributed to microglia^23^.

We first converted the raw spatial expression matrix by binning spots into hexbin pseudospots with a diameter of 40 μm and a center-to-center distance of 40 μm (tissue domains (TDs)), and retained only those with at least 30 human transcripts for further analysis. A total of 32,568 TDs were obtained across the cortical regions of the four samples, with 17,186 bins in the Lecanemab-treated samples and 15,382 from Lecanemab LALA-PG samples (Extended Data Fig. 2a,b). Each TD captured an average of 61.3 human genes and 74.4 unique molecular identifiers (UMIs; Extended Data Fig. 2c). We then performed a continuous differential expression (DE) analysis of cortical bins based on their distance from D54D2^+^ Aβ plaques, revealing that the expression of several genes substantially increases in function of proximity to plaques in the Lecanemab-treated mice, including *APOE, CTSD, SPP1, CD74* and other genes associated to antigen presentation (Fig. 1e). Notably, gene set enrichment analysis (GSEA) indicated a significant increase in the expression of pathways related to the lysosome (Fig. 1f and Extended Data Fig. 2d) and phagosome (Fig. 1g and Extended Data Fig. 2d), specifically in the Lecanemab-treated TDs. Interestingly, these gene sets were significantly downregulated in Lecanemab LALA-PG TDs in proximity to pathology (Fig. 1f,g and Extended Data Fig. 2e), suggesting that the Fc fragment is critical for the activation of these pathways in microglia near plaques. That said, we detected distinct transcriptomic effects induced by the Lecanemab LALA-PG treatment (Extended Data Fig. 2e,f), suggesting that the accumulation of nonfunctional antibody also modulates microglial activity near plaques. These data prompted further investigation into how the observed enhancements in phagocytosis and lysosomal functions by Lecanemab might affect plaque load.

### Lecanemab attenuates Aβ pathology through Fc-mediated microglial phagocytosis

**Fig. 2:**
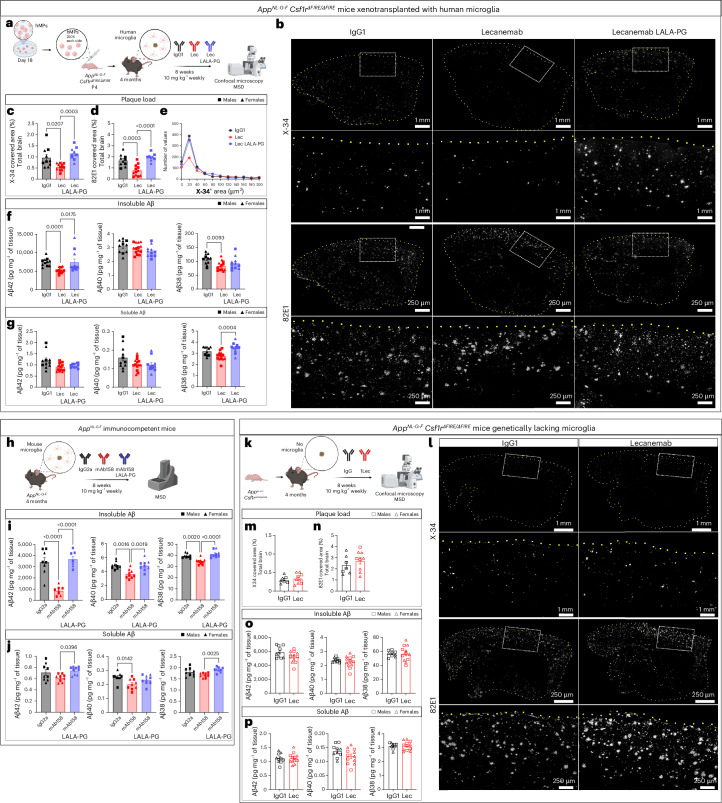
Lecanemab alleviates Aβ pathology by triggering effector functions in the microglia. **a**, *App*^*NL-G-F*^ *Csf1r*^*ΔFIRE/ΔFIRE*^ mice were xenotransplanted at P4 with human-derived microglial progenitors differentiated in vitro. Starting from 4 months of age, mice were treated for 8 weeks with IgG1, Lecanemab or Lecanemab LALA-PG (10 mg kg^−1^, weekly i.p. injections) and killed for subsequent analysis. **b**, Representative confocal images of plaques stained with X-34 or 82E1 in sagittal brain sections from mice treated with the indicated antibodies; scale bar = 1 mm; inset = 250 µm. **c**,**d**, Quantification of X-34 (**c**) and 82E1 (**d**) area expressed as percentage of the total section area; (**c**) Kruskal–Wallis test (*P* = 0.0003) and (**d**) one-way ANOVA (*P* < 0.0001; IgG1, *n* = 10 mice; Lec, *n* = 12 mice; Lec LALA-PG, *n* = 9 mice). **e**, Distribution of X-34^+^ plaques based on their area (*x* axis) shows that Lecanemab mainly affects small plaques; Anderson–Darling test, *P* < 0.0001 (IgG1 versus Lecanemab, *P* < 0.0003; Lecanemab versus Lecanemab LALA-PG, *P* < 0.0003; IgG1 versus Lecanemab LALA-PG, NS; two-sided Kolmogorov–Smirnov test; IgG1, *n* = 975 X-34^+^ plaques from 10 mice; Lec, *n* = 731 X-34^+^ plaques from 12 mice; Lec LALA-PG, *n* = 942 X-34^+^ plaques from 9 mice). To account for multiple comparisons (three in total), we applied a Bonferroni correction by multiplying the obtained *P* values by 3. **f**, MSD ELISA of Aβ42 (*P* = 0.0001, Kruskal–Wallis test followed by Dunn’s multiple comparison test), Aβ40 (NS, one-way ANOVA) and Aβ38 (*P* = 0.0117, one-way ANOVA followed by Bonferroni’s multiple comparisons test) levels in insoluble (GuHCl extractable) brain extracts (IgG1, *n* = 11 mice; Lec, *n* = 14 mice; Lec LALA-PG, *n* = 11 mice). **g**, MSD ELISA of Aβ42 (*P* = 0.0505, Kruskal–Wallis test), Aβ40 (NS, one-way ANOVA) and Aβ38 (*P* = 0.0005, one-way ANOVA followed by Bonferroni’s multiple comparisons test) levels in soluble (T-PER buffer extractable) brain extracts (IgG1, *n* = 10-11 mice; Lec, *n* = 14 mice; Lec LALA-PG, *n* = 11 mice). **h**, A cohort of immunocompetent *App*^*NL-G-F*^ mice was used to assess the impact of mAb158 (a mouse version of Lecanemab), its engineered LALA-PG variant and a control mouse IgG2a on Aβ levels by MSD. **i**, MSD ELISA of Aβ42 (*P* < 0.0001, one-way ANOVA followed by Bonferroni’s multiple comparisons test), Aβ40 (*P* = 0.0005, one-way ANOVA followed by Bonferroni’s multiple comparisons test) and Aβ38 (*P* < 0.0001, one-way ANOVA followed by Bonferroni’s multiple comparisons test) levels in insoluble brain extracts (IgG2a, *n* = 8–9 mice; mAb158, *n* = 8–9 mice; mAb158 LALA-PG, *n* = 7–9 mice). **j**, MSD ELISA of Aβ42 (*P* = 0.0355, one-way ANOVA followed by Bonferroni’s multiple comparisons test), Aβ40 (*P* = 0.0026, one-way ANOVA followed by Bonferroni’s multiple comparisons test) and Aβ38 (*P* = 0.0032, one-way ANOVA followed by Bonferroni’s multiple comparisons test) levels in soluble brain extracts (IgG2a, *n* = 8–9 mice; mAb158, *n* = 9 mice; mAb158 LALA-PG, *n* = 9 mice). **k**, A cohort of *App*^*NL-G-F*^ *Csf1r*^*ΔFIRE/ΔFIRE*^ mice was not xenotransplanted and used to assess if Lecanemab alters Aβ pathology in the absence of microglia. Panels **a**, **h** and **k** were created with BioRender.com. **l**, Representative confocal images for X-34 and 82E1 in sagittal brain sections from *App*^*NL-G-F*^ *Csf1r*^*ΔFIRE/ΔFIRE*^ mice treated with IgG1 or Lecanemab; scale bars = 1 mm, inset = 250 µm. **m**,**n**, Quantification of X-34 (**m**) and 82E1 (**n**) areas (unpaired two-sided *t*-tests, NS) expressed as percentage of the section area (IgG1, *n* = 7 mice; Lec, *n* = 9 mice). **o**, MSD ELISA of Aβ42, Aβ40 and Aβ38 levels in insoluble brain extracts (unpaired two-sided *t*-tests, NS; IgG1, *n* = 9–10 mice; Lec, *n* = 12 mice). **p**, MSD ELISA of Aβ42, Aβ40 and Aβ38 levels in soluble brain extracts (unpaired two-sided *t*-tests, NS; IgG1, *n* = 9–10 mice; Lec, *n* = 12 mice). Mean ± s.e.m. shown for each group and points represent individual animals. Square symbols, males; triangle, females. hMPs, human microglial progenitors; NS, not significant; ANOVA, analysis of variance. Source data

To assess the impact of Fc-mediated phagocytosis on Aβ plaque load, we treated a cohort of xenotransplanted *App*^*NL-G-F*^ *Csf1r*^*ΔFIRE/ΔFIRE*^ mice with Lecanemab, Lecanemab LALA-PG or human IgG1 control for 8 weeks (Fig. 2a). After the completion of the treatment (24 h after the last injection), we collected the brains (Fig. 2b) and used immunohistochemistry to analyze the impact of treatment on amyloid pathology. We quantified both β-sheeted amyloid aggregates using X-34 (Fig. 2c), and Aβ-peptides using a specific antibody (82E1; Fig. 2d). Lecanemab treatment significantly reduced plaque area compared to IgG1 or Lecanemab LALA-PG. Interestingly, when analyzing X-34^+^ plaque distribution, we observed that Lecanemab had the most pronounced effect on smaller plaques (Fig. 2e). Histological data were corroborated by the Meso Scale Discovery (MSD) platform, which revealed significantly reduced guanidine-extractable (insoluble) Aβ42 levels in Lecanemab-treated mice compared to those treated with IgG1 or Lecanemab LALA-PG (Fig. 2f). Notably, Aβ42 is the predominant Aβ species in *App*^*NL-G-F*^ mice^24^. A significant reduction in insoluble Aβ38 levels was also observed in Lecanemab-treated mice compared to IgG1-treated mice (Fig. 2f), while soluble Aβ38 levels were significantly reduced in Lecanemab-treated mice compared to those treated with Lecanemab LALA-PG (Fig. 2g). No changes were observed in insoluble Aβ40 levels (Fig. 2f,g). Our *App*^*NL-G-F*^ *Csf1r*^*ΔFIRE/ΔFIRE*^ mice lack a mature adaptive immune system to allow colonization of the human brain with xenografted human microglia. To demonstrate that Lecanemab’s effect on plaque load is not hampered by the presence of adaptive immune cells, we repeated the experiments in a cohort of immunocompetent *App*^*NL-G-F*^ mice treated with mAb158 (a mouse version of Lecanemab), its engineered LALA-PG variant and a control mouse IgG2a (Fig. 2h). After 8 weeks of treatment, mAb158 significantly decreased Aβ levels as measured by MSD (Fig. 2i,j), whereas the LALA-PG mutation eliminated the antibody-mediated Aβ clearance effect in this model with an intact mouse immune system. Finally, we demonstrated the essential role of microglia in Lecanemab-mediated plaque clearance as *App*^*NL-G-F*^ *Csf1r*^*ΔFIRE/ΔFIRE*^ mice that did not receive xenotransplantation (Fig. 2k) showed no impact from Lecanemab on plaque load (Fig. 2l–p). Collectively, these findings demonstrate that Lecanemab attenuates Aβ pathology in vivo through Fc-mediated microglial effector functions.

**Fig. 3:**
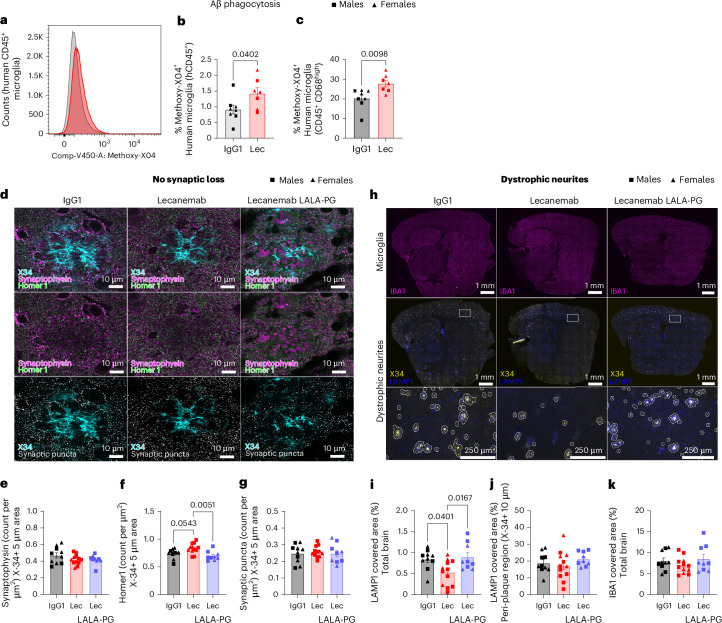
Lecanemab induces phagocytosis of Aβ and alleviates downstream consequences of Aβ pathology through Fc-mediated microglial effector functions. **a**, Flow cytometry analysis of Methoxy-X04^+^ cells within the hCD45^+^ population (microglia) after 8 weeks of Lecanemab (red) or IgG1 (black) treatment. The *x* axis represents Methoxy-X04 fluorescence intensity, while the *y* axis shows the number of cells. The overlaid histograms (black and red) highlight differences in Methoxy-X04 staining levels. **b**, Percentage of X04^+^ microglia (CD45^+^) isolated from IgG1 or Lecanemab-treated mice (unpaired two-sided *t*-test; IgG1, *n* = 8 mice; Lec, *n* = 7 mice). **c**, Percentage of X04^+^ microglia (CD45^+^, CD68^high^) isolated from IgG1 or Lecanemab-treated mice (unpaired two-sided *t*-test; IgG1, *n* = 8 mice; Lec, *n* = 7 mice). **d**, Representative super-resolution confocal images of synaptic loss surrounding X-34^+^ plaques in IgG1-treated, Lecanemab-treated and Lecanemab LALA-PG-treated xenotransplanted mice. Synaptic puncta were defined as synaptophysin (magenta) and Homer1 (green) immunoreactive puncta (white) around X-34^+^ plaques (cyan). Scale bar = 10 µm. **e**–**g**, Quantification of synaptophysin (one-way ANOVA, NS; **e**), Homer1 (one-way ANOVA, *P* = 0.044; **f**) and synaptic puncta (one-way ANOVA, NS; **g**), in peri-plaque area (defined as 5 µm from the X-34 edges; IgG1, *n* = 10 mice; Lec, *n* = 12 mice; Lec LALA-PG, *n* = 9 mice). **h**, Representative confocal images for IBA1 (magenta), X-34 (yellow) and LAMP1 (blue) in sagittal brain sections from *App*^*NL-G-F*^ *Csf1r*^*ΔFIRE/ΔFIRE*^ mice xenotransplanted with human-derived microglia and treated with IgG1, Lecanemab and Lecanemab LALA-PG; scale bars = 1 mm, insets = 250 µm. The area of LAMP1 was assessed in the 10-µm rings surrounding X-34 (peri-plaque region) and then divided by the area of the brain section or the peri-plaque area. **i**,**j**, Quantification of LAMP1 area expressed as percentage of the total brain section (**i**) and the peri-plaque area (**j**); (**i**) one-way ANOVA (*P* = 0.0091) and (**j**) one-way ANOVA (NS; IgG1, *n* = 10 mice; Lec, *n* = 12 mice; Lec LALA-PG, *n* = 9 mice). **k**, Quantification of IBA1 area expressed as percentage of the total brain section (Kruskal–Wallis test, NS; IgG1, *n* = 10 mice; Lec, *n* = 12 mice; Lec LALA-PG, *n* = 9 mice). Mean ± s.e.m. shown for each group and points represent individual animals. Square symbols, males; triangle, females. Source data

**Extended Data Fig. 3:**
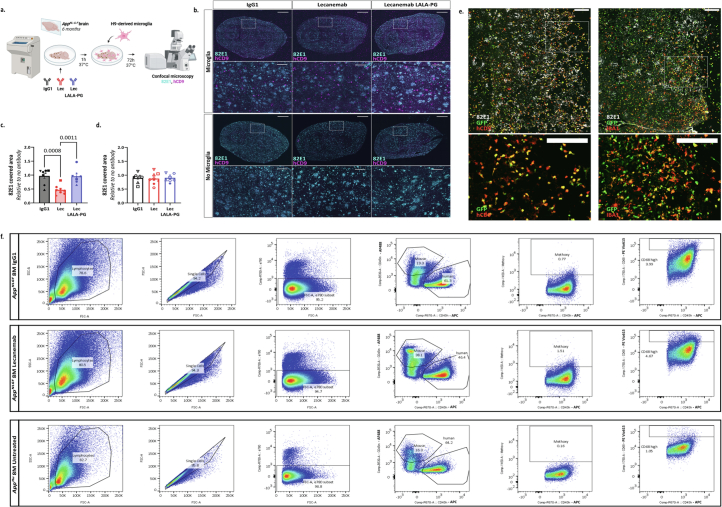
Lecanemab induces plaque clearance in an in vitro assay. **a**, Schematic representing the plaque clearance assay paradigm to study Aβ clearance in response to Lecanemab. Sagittal cryosections from App^NL-G-F^ mice at 6 months of age were pre-incubated for 1 h with IgG1, Lecanemab or Lecanemab LALA-PG. We then added human-derived microglial cells to the cryosections and, after 3 days, quantified the area covered by the pan-Aβ antibody 82E1. Panel **a** was created with BioRender.com. **b**, Representative confocal images of 82E1 (Aβ, cyan) and CD9 (human microglia, magenta) immunoreactivity in App^NL-G-F^ brain cryosections after incubation with antibodies as indicated, in presence or absence of human microglia. Scale bar = 1 mm; inset = 200 µm. **c**, Quantification of 82E1 covered area (relative to no antibody condition) in sections plated with human microglia; one-way ANOVA (p = 0.0003) (n = 8 independent experiments). Mean ± s.e.m. shown for each group and points represent independent experiments being each the average of 2–6 cryosections. **d**, Quantification of 82E1 covered area (relative to no antibody condition) in sections nonplated with human microglia; one-way ANOVA (NS) (n = 8 independent experiments). Mean ± s.e.m. shown for each group and points represent independent experiments being each the average of 2–6 cryosections. **e**, Colocalization GFP^+^ microglia with hCD9 and IBA1. Scale bar = 200 µm. Microglia derived from the H9 stem-cell line stably expressing GFP were plated on sagittal cryosections from 6-month-old App^NL-G-F^ mice. After 3 days, cells were fixed to assess GFP^+^ expression of hCD9 and IBA1. The majority of GFP^+^ cells co-express hCD9 (left), while not all GFP^+^ cells are IBA1^+^ (right). This comparison was performed in two independent experiments with similar results. **f**, Representative FACS plots of methoxy-X04⁺ human microglia from 8-month-old App^NL-G-F^ mice injected with IgG1 (top) or Lecanemab (middle), along with untreated App^Hu^ mice as a negative control (bottom). Live human cells were identified based on CD11b⁺ hCD45⁺ expression, with the negative control used to establish the gating for methoxy-X04⁻ cells. Methoxy-X04⁺ cells were quantified as a percentage of hCD45⁺ human microglia and within the CD68^high⁺ population (Fig. 3a–c). FACS, fluorescence-activated cell sorting. Source data

We then evaluated the impact of Lecanemab on microglial phagocytosis of Aβ fibrils. In an in vitro assay using cryosections from *App*^*NL-G-F*^ mouse brains (Extended Data Fig. 3a), sections were incubated for 1 h with control IgG1, Lecanemab or Lecanemab LALA-PG^14^. Subsequently, human-derived microglial cells^16^ were added and, after 3 days, amyloid plaque area was quantified using the pan-Aβ antibody 82E1 (Extended Data Fig. 3b). Lecanemab treatment sections exhibited a significantly reduced Aβ-plaque load relative to those exposed to control IgG1 or Lecanemab LALA-PG (Extended Data Fig. 3c). Notably, in the absence of microglia, no plaque clearance was observed (Extended Data Fig. 3d), confirming that Lecanemab facilitates microglia-mediated Aβ clearance in vitro. To validate these findings in vivo, a cohort of *App*^*NL-G-F*^ mice xenotransplanted with human microglia was treated with either Lecanemab or IgG1 from 6 to 8 months of age. After the treatment, the mice received an intraperitoneal injection of Methoxy-X04, a fluorescence probe that crosses the blood–brain barrier to stain Aβ^25^. Three hours after injection, human CD45⁺ microglia were collected and the proportion of Methoxy-X04⁺ cells was quantified by flow cytometry (Fig. 3a and Extended Data Fig. 3f). Lecanemab treatment resulted in a significant increase in Aβ uptake by hCD45⁺ microglia, particularly within the CD68^high^ subset (Fig. 3b,c).

Given that increased phagocytosis may be detrimental if nonspecific—for instance, by leading to the removal of synapses in the proximity of plaques^9^—we assessed the density of the presynaptic marker Synaptophysin, the postsynaptic marker Homer1, and their overlap (synaptic puncta) in the peri-plaque area (defined as 5 µm from the X-34 edges; Fig. 3d). Synaptophysin (Fig. 3e) and synaptic puncta densities (Fig. 3g) remained unchanged, and we found a small but significant increase in postsynaptic density in Lecanemab-treated mice compared to IgG1-treated and Lecanemab LALA-PG-treated animals (Fig. 3f). These findings suggest that Lecanemab-induced phagocytosis is specific to Aβ. We further investigated the downstream effects of reduced plaque load on Aβ-related pathologies. Lecanemab treatment significantly decreased neuritic pathology, as indicated by LAMP1 staining across the total brain area (Fig. 3h,i). However, the ratio of LAMP1-positive area to amyloid plaque load was unchanged (Fig. 3j), suggesting that the reduction in dystrophic neurites is an indirect consequence of decreased plaque load.

### Lecanemab remodels microglial transcriptome to activate clearance pathways

**Fig. 4:**
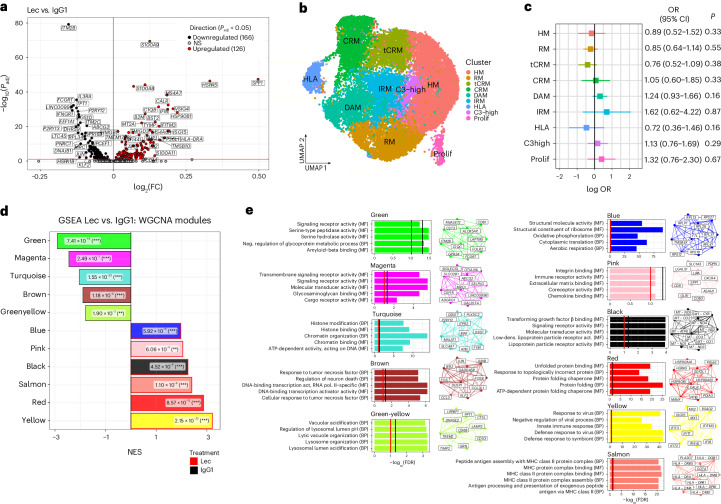
Transcriptomic changes in human microglia treated with Lecanemab. **a**, Volcano plots showing a gene expression comparison between Lecanemab-treated and IgG1-treated human microglia (*n* = 6 mice per condition). The number of significant genes per condition is reported in brackets. *P*_adj_ threshold < 0.05 (two-sided Wilcoxon rank-sum test, *P* values adjusted with Bonferroni correction based on the total number of genes in the dataset, NS). **b**, UMAP plot visualizing 22,420 (10,850 IgG1, 11,570 Lecanemab) human microglial cells after the removal of macrophages. Cells are colored according to clusters identified. The assignment of different clusters to distinct cell types or states is based on the previous experimental data from our laboratory^14^. **c**, OR and 95% CI for the differential abundance of cell states between Lecanemab-treated (*n* = 6 mice) and IgG1-treated group (*n* = 6 mice) using MASC analysis. Points indicate the estimated OR, with horizontal lines representing the 95% CI. Two-sided tests were performed, and exact *P* values are shown. No significant changes in the proportion of cell states are detected between IgG1 and Lecanemab-treated microglia. **d**, NES of significantly enriched (*P*_adj_ < 0.05) WGCNA modules between IgG1 and Lecanemab-treated cells, as identified by GSEA with *P*_adj_ values indicated. Two-sided *P* values were adjusted using the BH correction (**P_adj_ < 0.01, ***P_adj_ < 0.001). **e**, Functional annotations based on GO pathway analyses (MF and BP) using an overrepresentation analysis (one-sided hypergeometric test). Enrichment for a given ontology is shown by *q* score, with thresholds indicated by red line (*q* < 0.1) and black line (*q* < 0.05). The top ten hub genes are shown based on the module eigengene-based connectivity (kME). *P* values were adjusted using the BH correction. OR, odds ratio; CI, confidence interval; MF, molecular function; BP, biological process; MASC, mixed-effects modeling association of single cells. Source data

**Extended Data Fig. 4:**
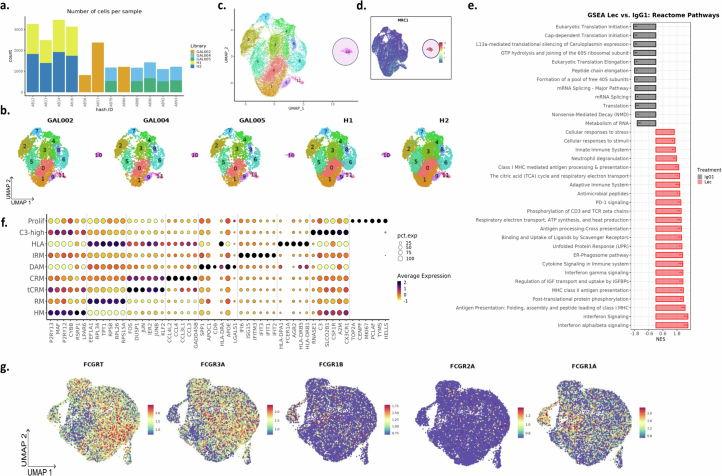
Preparation of the datasets for analysis and cell type/state annotations. **a**,**b**, Overview of the cDNA libraries used in scRNA-seq experiments (GAL002, GAL004, GAL005, H1 and H2), including the number of cells per sample (**a**) and the distribution of cells on the UMAP across different libraries after data integration (**b**). **c**, The UMAP plot of the 22,841 single xenografted human cells that passed quality control colored by clusters. **d**, UMAP plot colored by the expression level of *MRC1*, a macrophage marker. Notably, cluster 10, identified as the macrophage cluster, was removed from further analysis. **e**, Reactome pathway enrichment terms for significantly downregulated (black) and upregulated genes (red) ranked by p-value upon Lecanemab treatment (*: p.adj < 0.05, **: p.adj < 0.01, ***: p.adj < 0.001). Two-sided p-values were adjusted using the Benjamini–Hochberg (BH) correction. **f**, Top six most differentially expressed genes in each cell state. The dot size represents the percentage of cells within the given cluster expressing the gene. The dot color represents the scaled and normalized average expression of the gene. **g**, UMAP plots colored by the level of expression of *FCGRs* (encoding FcγRs) expressed by human microglia.

Microglia clear amyloid plaques effectively only when Lecanemab is present, suggesting that Fc engagement modifies their functionality. To investigate the mechanisms underlying plaque clearance with greater resolution, we performed a single-cell transcriptomic profiling of human microglia using the 10x Genomics platform, achieving deeper gene expression coverage than through spatial transcriptomics. Because our data indicated that Lecanemab LALA-PG strongly accumulates on Aβ-plaques and affects microglial responses (Fig. 1b,c), we compared microglia treated with Lecanemab to those treated with a control IgG1. After quality control and removal of macrophages (Extended Data Fig. 4a–d), a differential gene expression analysis between IgG1-treated and Lecanemab-treated human microglia revealed approximately 300 significantly differentially expressed genes (Fig. 4a). Consistent with our spatial transcriptomics results, upregulated genes were enriched for terms associated with the phagosome pathway and antigen presentation (Extended Data Fig. 4e). Additionally, we identified genes enriched for processes related to interferon response, metabolism and the unfolded protein response (Extended Data Fig. 4e).

**Fig. 5:**
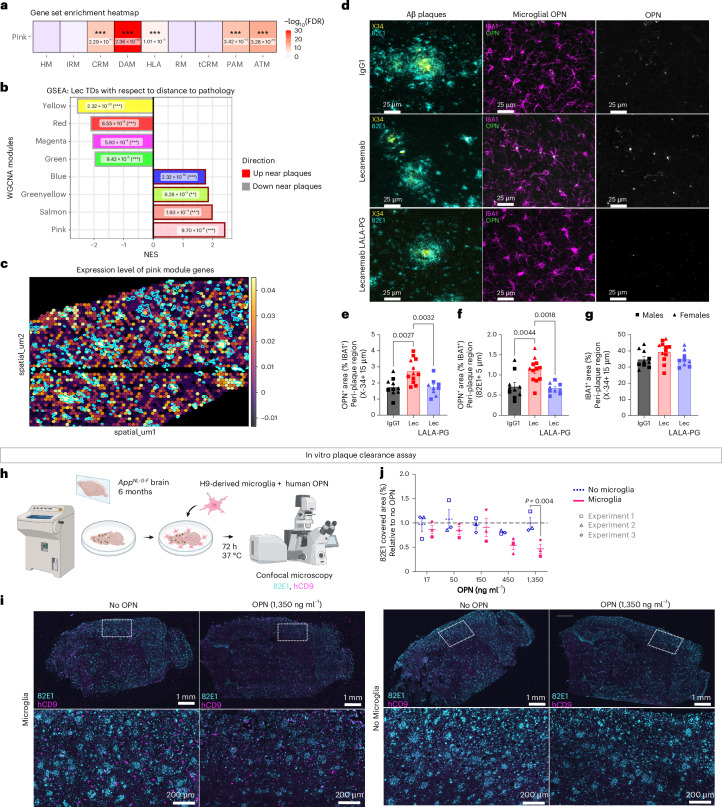
OPN/SPP1, one of the main factors induced by Lecanemab treatment, promotes Aβ clearance. **a**, Heatmap displaying the significance of enrichment of marker genes of previously reported microglial cell states^16,26,27^ in the pink WGCNA module, as assessed by a one-sided hypergeometric overlap test. Color intensity reflects enrichment significance, represented as −log_10_(FDR), with darker red indicating stronger enrichment. Respective BH *P*_adj_ values for the enrichments are specified. The list of shared genes with significantly enriched states is outlined in Supplementary Table 3 (***P_adj_ < 0.001). **b**, NES of WGCNA modules in Lecanemab TDs (40-μm hexbin pseudospots) with respect to distance to pathology, as identified by GSEA, performed on the DE analysis in Fig. 1e, with *P*_adj_ values indicated. Two-sided *P* values were adjusted using the BH correction (**P_adj_< 0.01, ***P_adj_ < 0.001). **c**, Cortical TDs in Lecanemab-treated mice colored based on their relative expression of genes belonging to the pink module (purple, low expression; yellow, high expression) and overlayed with plaque ROIs (white). ESs were obtained using Scanpy’s ‘score_genes()’ function. Please note the significant enrichment of the module in TDs close to Aβ plaques, as quantified in **b**. **d**, Representative confocal images of cortical X-34^+^ and 82E1^+^ plaques surrounded by OPN^+^ microglia in IgG1-treated, Lecanemab-treated and Lecanemab LALA-PG-treated mice. Scale bar = 25 µm. **e**,**f**, Quantification of the area of OPN^+^ area within IBA1^+^ cells around X-34^+^ (**e**) and 82E1^+^ plaques (**f**) in IgG1-treated and Lecanemab-treated mice; (**e**) one-way ANOVA (*P* = 0.0008) and (**j**) one-way ANOVA (*P* = 0.0007; IgG1, *n* = 10 mice; Lec, *n* = 12 mice; Lec LALA-PG, *n* = 9 mice). **g**, Quantification of the area covered by IBA1^+^ cells around X-34^+^ plaques; one-way ANOVA (NS) (IgG1, *n* = 10 mice; Lec, *n* = 12 mice; Lec LALA-PG, *n* = 9 mice). **h**, Schematic representation of the in vitro plaque clearance assay paradigm used to study Aβ clearance in response to OPN stimulation. Human-derived microglial cells were plated onto sagittal cryosections from 6-month-old *App*^*NL-G-F*^ mice, followed by the treatment with increasing concentrations of human OPN (17, 50, 150, 450 and 1,350 ng ml^−1^). After 3 days, Aβ plaque coverage was quantified using the pan-Aβ antibody 82E1. Panel **h** was created with BioRender.com. **i**, Representative confocal images of 82E1 (Aβ, cyan) and CD9 (human microglia, magenta) immunoreactivity in *App*^*NL-G-F*^ brain cryosections after OPN stimulation. Scale bar = 1 mm; inset = 200 µm. **j**, Quantification of 82E1 covered area (relative to no OPN) in sections plated with or without human microglia; modified chi-squared method (*n* = 3 independent experiments; *P* = 0.03). Graphs show mean ± s.e.m. and points represent individual animals (**e**–**g**) or independent experiments, with each being the average of one to four cryosections (**j**). Square symbols, males; triangles, females. Source data

**Extended Data Fig. 5:**
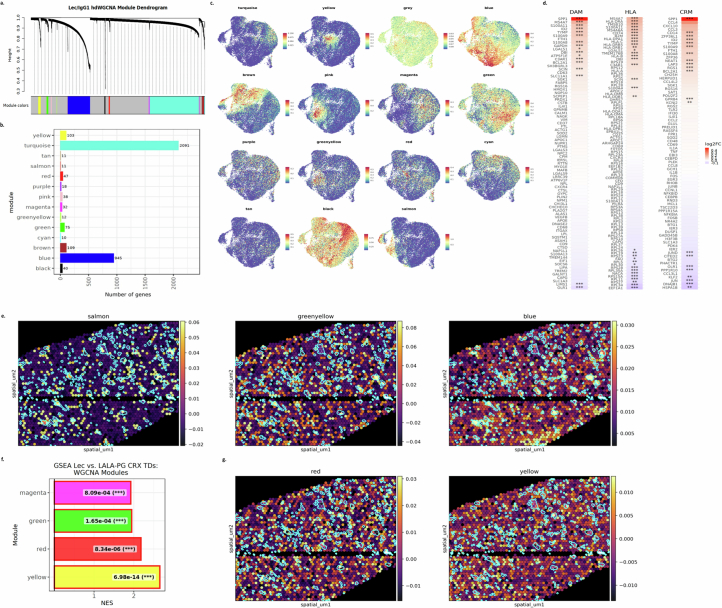
Lecanemab affects pathways linked to unfolded protein response, cell metabolism, antigen presentation and interferon response. **a**, Weighted gene co-expression network analysis (WGCNA) cluster dendrogram groups genes measured across IgG1- and Lecanemab-treated cells into 14 distinct modules, defined by dendrogram branch cutting. An additional ‘unassigned’ gray module was identified but discarded from subsequent analyses. **b**, Number of genes belonging to identified WGCNA modules. **c**, UMAP plots colored by the combined level of expression of each WGCNA module. **d**, Heatmaps displaying the log fold changes (LFCs) of microglial cell state markers (DAM, HLA, CRM^16^) in differential expression between Lecanemab-treated and IgG1-treated microglia. Color intensity reflects the magnitude and direction of differential expression, asterisks denote statistical significance (*: FDR < 0.05, **: FDR < 0.01, ***: FDR < 0.001). **e**, Cortical tissue domains (TDs) in Lecanemab-treated mice colored based on their relative expression of genes belonging to the salmon (left), greenyellow (middle) and blue (right) modules (purple: low expression, yellow: high expression) and overlayed with plaque ROIs (outlined in cyan). Enrichment scores were obtained using Scanpy’s *score_genes()* function. Note the significant enrichment of the module in bins close to Aβ plaques, as quantified in Fig. 2b. **f**, NES of significantly enriched (p.adj < 0.05) weighted gene co-expression network analysis (WGCNA) modules between Lecanemab and Lecanemab LALA-PG-treated TDs in cortical regions, as identified by GSEA with p.adj values indicated. Two-sided p-values were adjusted using the Benjamini–Hochberg (BH) correction (***: p.adj < 0.001). **g**, Cortical TDs in Lecanemab-treated mice colored based on their relative expression of genes belonging to the red (left) and yellow (right) modules (purple: low expression, yellow: high expression) and overlayed with plaque ROIs (outlined in cyan).

Although we observed changes in gene expression levels, their magnitude is relatively subtle. Furthermore, Lecanemab’s efficacy does not appear to be driven by significant shifts in specific cell state populations, which primarily reflect broad transcriptional changes, such as the disease-associated microglia (DAM)/human leukocyte antigen (HLA) state (Fig. 4b,c and Extended Data Fig. 4f). This insight prompted us to investigate whether the gene set induced by Lecanemab is more specifically targeted. To do so, we performed weighted gene coexpression network analysis (WGCNA) and identified 14 modules of coexpressed genes (Extended Data Fig. 5a–c and Supplementary Table 1). GSEA revealed that five modules were significantly downregulated and six modules were significantly upregulated after the Lecanemab treatment (Fig. 4d). Among the upregulated modules (Fig. 4e and Supplementary Table 2), the yellow module was enriched for interferon genes, the red module for unfolded protein and protein folding genes, the salmon module for antigen presentation genes, the black module for mitochondrial and immune signaling genes and the blue module for metabolism genes, particularly those involved in oxidative phosphorylation and aerobic respiration. The pink module, although not associated with a specific functional signature in gene ontology (GO) databases, was enriched for *SPP1*, *LGALS1, CTSD* and *ITGAX*, core genes of DAM/HLA, as well as the protective axon-tract-associated microglia (ATM) and proliferative-region-associated microglia (PAM) identified during early microglia development^26,27^ (Fig. 5a, Extended Data Fig. 5d and Supplementary Table 3). Notably, *SPP1* (encoding osteopontin (OPN)) is also the most strongly upregulated gene in both our single-cell RNA sequencing (scRNA-seq) and spatial DE analyses. *SPP1* is implicated in phagocytosis^26,28^ and has been linked to a protective response in developing microglia^26^. Although *SPP1* and other pink module genes are markers of DAM/HLA^16^, which typically accumulate around amyloid plaques without clearing them, our findings suggest that Lecanemab induces even higher expression of these genes, which activate clearance programs that are not fully engaged in DAM/HLA. Finally, in contrast to reports that Aducanumab—another anti-Aβ antibody—induces canonical inflammatory genes (*Tnf*, *Il1b*, *Nfkb*)^6,8^, Lecanemab-treated microglia exhibited reduced expression of the inflammatory brown module (Fig. 4e and Supplementary Table 2).

We then used our functional WGCNA modules generated on the scRNA-seq data to interpret changes in the spatial transcriptomic data relative to plaque proximity. Notably, two key modules identified as upregulated in the single-cell GSEA, the yellow module (interferon-related) and the red module (unfolded protein response), do not seem to be spatially associated with plaques, although significantly enriched globally in the Lecanemab TDs compared to the Lecanemab LALA-PG TDs (Extended Data Fig. 5f,g). On the other hand, the pink module (enriched for *SPP1*) was the most significantly enriched in plaque-associated TDs (Fig. 5b,c), followed by modules associated with antigen presentation (salmon), lysosome (green-yellow) and metabolism (blue; Extended Data Fig. 5e). Consistent with our transcriptomic data, OPN expression was elevated in IBA1^+^ microglia surrounding X-34^+^ and 82E1^+^ plaques in Lecanemab-treated mice compared to those treated with IgG1 or Lecanemab LALA-PG (Fig. 5d–f). However, no significant increase in IBA1^+^ area around X-34^+^ plaques was observed (Fig. 5g), suggesting that the rise in OPN reflects an increased expression per cell rather than a higher number of OPN-expressing cells. This observation aligns with our results from the scRNA-seq DE analysis (Fig. 4a).

### OPN-driven Aβ clearance in Lecanemab-treated microglia

The proximity of OPN^+^ cells to amyloid plaques suggests that Lecanemab restores protective phagocytic functions in human microglia near Aβ deposits. To test this, we used our in vitro plaque clearance system (Extended Data Fig. 3a–e) and stimulated human-derived microglia with increasing concentrations of human OPN (Fig. 5h,i). Remarkably, at the highest concentration tested, OPN stimulation significantly decreased the area covered by the pan-Aβ antibody 82E1 (Fig. 5j), demonstrating that OPN, one of the main factors induced by Lecanemab, promotes Aβ clearance.

## Discussion

Lecanemab stands as the most successful Aβ-plaque-clearing antibody in clinical use^2,13^. Our study demonstrates that its efficacy critically depends on the presence of microglia and the engagement of Fc effector functions, which activate a targeted amyloid-clearing program in these cells. Transcriptomic and functional analyses reveal that Lecanemab induces a distinct program in microglia that enhances phagocytosis and lysosomal activity without triggering the synaptophagy observed in other studies^9^. This selective activation correlates with a reduction in neuritic pathology, potentially underpinning the modest clinical improvements observed with Lecanemab.

Among the Lecanemab-induced upregulated genes, several are highly relevant to AD. For instance, the *MS4A* gene family, including *MS4A6A*, is strongly associated with AD risk and functions upstream of TREM2 (ref. ^29^). Upregulation of *HSPA5*, an ER stress marker, may indicate enhanced Aβ uptake in Lecanemab-treated microglia, triggering the unfolded protein response^30^. Moreover, *SPP1* was the most strongly upregulated gene in both our scRNA-seq and spatial transcriptomic differential gene expression analyses. It is important to note, however, that the overall magnitude of changes in expression levels is relatively limited, which, we postulate, may be due to the fact that the changes in our microglia are primarily localized to those in close proximity to the plaques. For this reason, despite acknowledging the potential biases of cell-level DE analyses, we believe a pseudobulk approach, which has been shown to better control for false discoveries^31^, would mask changes in distinct cell populations.

Nevertheless, despite the subtlety of these changes, our experimental work clearly demonstrates that Lecanemab confers a functional ability to clear plaques. To further extend our investigation of the transcriptional programs associated with this ability, we performed WGCNA, which corroborated our GSEA by revealing that Lecanemab-induced genes cluster into distinct modules associated with the interferon response, antigen presentation, metabolism and the unfolded protein response. Strikingly, when we leveraged our functional WGCNA modules to analyze spatial transcriptomic changes relative to plaque proximity, we identified the pink module (enriched for *SPP1*) as significantly enhanced around Aβ plaques in the Lecanemab-treated samples, further supporting our scRNA-seq findings. *SPP1*, a key hub gene in this module, is a well-established marker of DAM/HLA^16^, cells that accumulate around amyloid plaques without effectively clearing them. Its further induction by Lecanemab suggests the activation of clearance pathways that extend beyond the conventional DAM state. This is supported by our findings that exogenous OPN enhances the amyloid clearance capacity of human microglia. Future studies should investigate whether additional genes and pathways within the Lecanemab-induced pink module contribute to amyloid clearance, and whether this transcriptional profile represents an amplification of known DAM/HLA microglia cell states or defines a distinct microglial phenotype with enhanced amyloid-clearing capacity. In any event, phagosome/phagocytosis emerged as one of the top pathways in both scRNA-seq and spatial GSEA analyses, and we functionally validated this signature by demonstrating that Lecanemab enhances Aβ phagocytosis in vivo and in vitro. Collectively, these findings indicate that a limited set of genes is sufficient to reprogram microglia for efficient amyloid clearance.

Our work highlights the unique advantages of our xenograft human/mouse chimeric model, which enables direct in vivo evaluation of the unmodified human antibody on human microglia. Although the *Rag2*^*−/−*^ background necessary to prevent graft rejection precludes analysis of adaptive immunity, parallel experiments using a mouse variant of Lecanemab in immune-competent animals confirmed that amyloid clearance is not qualitatively different in the presence of adaptive immunity. This is particularly important given the distinct responses of human versus mouse microglia to amyloid plaques and the low sequence conservation of key AD risk genes like *MS4A6A*^16^ between species.

It is tempting to speculate that differences in the clinical efficacy of anti-Aβ antibodies may, in part, reflect their distinct impacts on microglial inflammatory responses. For example, while Aducanumab has been associated with robust pro-inflammatory activation^8^, our data suggest that Lecanemab elicits a more restrained immune activation, which may underlie its relatively greater clinical benefit. This contrast is intriguing but should be interpreted with caution, as prior studies were conducted in mouse microglia. In our study, Lecanemab-treated microglia upregulated interferon-responsive genes but did not broadly induce classical pro-inflammatory cytokines. Interestingly, interferon signaling has been implicated in brain endothelial dysfunction in AD^32^, suggesting that this pathway contributes to amyloid-related imaging abnormalities—the most impactful adverse events associated with Lecanemab. However, a key limitation of our model is its reduced capacity to capture vascular pathology or blood–brain barrier dysfunction, as *App* knock-in mice models do not show substantial vascular defects at the studied age^33^.

Given the central role of inflammation in immunotherapy, deciphering how Fc receptor-mediated microglial activation shapes responses in other central nervous system cell types remains a critical avenue for future research. Our findings also raise the possibility that distinct FcγRs expressed by microglia (Extended Data Fig. 4g and Supplementary Table 4) may differentially mediate beneficial versus detrimental effects of antibody treatment. Alternatively, activation of Fc or complement receptors on border-associated macrophages by the Fc moiety of Lecanemab could also trigger harmful vascular responses^34^. Finally, Lecanemab may reduce peripheral immune cell infiltration by dampening central nervous system inflammation, potentially contributing to its attenuation of pathology^35,36^. Dissecting these mechanisms will be essential to clarify the balance between therapeutic efficacy and adverse effects of anti-Aβ immunotherapy.

While we cannot rule out the possibility that other anti-Aβ antibodies differentially influence microglia by binding to distinct Aβ species^13^, our findings support the notion that the precise nature of the Aβ protofibrils^1^ targeted by Lecanemab is less critical than its ability to engage amyloid plaques and then microglia through its Fc moiety. This may explain the comparable clinical impact of Donanemab, another FDA-approved antibody that targets a pyroglutamate-modified amyloid peptide in the amyloid plaque itself^3^. Effective amyloid clearance may be achieved if the antibody binds amyloid fibrils sufficiently to correctly position its Fc domain for microglial activation. This insight opens new avenues for therapeutic innovation, including the development of small compounds linked to Fc fragments or the engineering of antibodies with enhanced effector functions and reduced complement activation, an approach extensively used in other medical fields^37^, to improve antibody treatment outcomes in AD.

## Methods

### Antibodies: design, production and quality control

Variable domain amino acid sequences for Lecanemab were retrieved from the KEGG DRUG database (Supplementary Table 5). Heavy and light chains were cloned into a single human IgG1 expression vector (pTRIOZ-hIgG, InvivoGen). For Lecanemab, the production was initially done in-house, then outsourced to GenScript. Synthetic genes encoding for the respective variable domains, preceded by the mouse Ig heavy leader signal (Twist Biosciences), were cloned into pTRIOZ-hIgG (Invivogen). The VL domain was cloned using the restriction enzymes AscI/BsiWI, and the VH domain using AgeI/NheI. The antibody encoding open reading frames were sequence confirmed by Sanger sequencing (Eurofins). Plasmid DNA was delivered to CHO cells (Thermo Fisher Scientific, A29127) by transient transfection according to the manufacturer’s protocol (CHOgro High Yield Expression System; Mirus Bio, MIR 6270). Transfected CHO cells were cultured for 14 days in suspension on agitation at 32 °C. Two weeks after transfection, cell supernatants were collected and incubated overnight at 4 °C with AmMag Protein A Magnetic Beads (GenScript, L00939). The beads were collected using a magnetic separation rack and targeted antibodies were separated using the AmMag SA Plus system (GenScript, L01013). To abolish Fc effector function, the heavy chain of Lecanemab was designed to include the P329G substitution combined with L234A and L235A (LALA-PG). The light chain was the same as for Lecanemab (Supplementary Table 5). Production was outsourced to GenScript. As a control, we used the human IgG1 isotype control (Imtec Diagnostics, LT9005). Production of the mouse antibodies (mAb158 and mAb158 LALA-PG) was outsourced to GenScript. The amino acid sequence mAb158 was retrieved from patent US 8025878 B2, seq IDs 115 and 116. The LALA-PG mutations were designed according to the details discussed in ref. ^15^. As a control, we used the mouse IgG2a isotype control (Leinco, P381). The purity of the antibodies was estimated to be above 75% by densitometric analysis of the Coomassie Blue-stained SDS–PAGE gel under nonreducing conditions. Binding to Aβ1-42 (rPeptide, A-1163-2) was confirmed by ELISA and Dot Blot as performed in refs. ^38,39^.

### Human microglial progenitors and xenotransplantation

Human embryonic stem cells WA09 (H9, female), obtained from WiCell Research Institute (WA09; RRID, CVCL_9773), were cultured on Matrigel (VWR International, BDAA356277) with E8‑Flex medium (Thermo Fisher Scientific, A2858501) at 37 °C and 5% CO_2_. At 70–80% confluence, colonies were dissociated with Accutase (Sigma-Aldrich, A6964) and aggregated as embryoid bodies in U‑bottom 96‑well plates (10,000 cells per well) in mTeSR1 (STEMCELL Technologies, 15883465) plus BMP4 (50 ng ml^−^^1^), VEGF (50 ng ml^−^^1^) and SCF (20 ng ml^−^^1^) for 4 days. Embryoid bodies were transferred to six‑well plates in X‑VIVO medium (Lonza, 02-060Q) plus SCF (50 ng ml^−^^1^), M‑CSF (50 ng ml^−^^1^), IL‑3 (50 ng ml^−^^1^), FLT3 (50 ng ml^−^^1^) and TPO (5 ng ml^−^^1^) for 7 days, then switched to X‑VIVO plus FLT3 (50 ng ml^−^^1^), M‑CSF (50 ng ml^−^^1^) and GM‑CSF (25 ng ml^−^^1^) on day 11. Floating microglial precursors were collected on day 18 and resuspended in 1× DPBS (Gibco, 14190-144) at 2.5 × 10^5^ cells per µl. P4 pups received 5 × 10^5^ cells intracerebrally as described in refs. ^16,40,41^. Cytokines were from PeproTech. Additional collections were obtained by returning embryoid bodies to X‑VIVO with FLT3, M‑CSF and GM‑CSF.

### Mice and husbandry

All mice were housed in a specific pathogen-free facility under a 14-h light/10-h dark cycle, at an ambient temperature of 21 °C and 40–60% humidity, in groups of two to five animals, with food and water provided ad libitum. All experiments were conducted according to the protocols approved by the local Ethical Committee for Laboratory Animals of the KU Leuven (government license, LA1210579; ECD projects P125/2022 and P132/2022), following the country and European Union guidelines.

*App*^*NL-G-F*^ mice (C57BL/6 background; strain *App*^*tm3.1Tcs+*^; discussed in ref. ^24^, RIKEN; RRID, IMSR_RBRC06344) were used. These mice express amyloid precursor proteins (APP) at endogenous levels with a humanized Aβ sequence carrying the Swedish (NL, K670_M671delinsNL), Arctic (G, E693G) and Iberian (F, I716F) familial AD-causing mutations.

*Rag2*^*tm1.1Flv*^; *Csf1*^*tm1(CSF1)Flv*^; *Il2rg*^*tm1.1Flv*^; *App*^*tm3.1Tcs*^; *Csf1R*^*em1Bdes*^ mice (mixed C57BL/6, Balb/c background; named *App*^*NL-G-F*^ *Csf1r*^*ΔFIRE/ΔFIRE*^ mice) were generated in-house at KU Leuven as described in refs. ^17,18^. Briefly, homozygous mouse oocytes from *Rag2*^*tm1.1Flv*^; *Csf1*^*tm1(CSF1)Flv*^; *Il2rg*^*tm1.1Flv*^; *App*^*tm3.1Tcs*^ crosses were micro-injected with reagents targeting the FMS-intronic regulatory sequence (FIRE sequence) in the intron 2 of the mouse *Csf1R* gene^18^. Ribonucleoproteins containing 0.3-μM purified Cas9HiFi protein 0.3-μM crRNA (5’GTCCCTCAGTGTGTGAGA3’ and 5’CAATGAGTCTGTACTGGAGC3’) and 0.3-μM *trans*-activating crRNA (Integrated DNA Technologies) were injected into the pronucleus of 120 embryos by the CBD Mouse Expertise Unit of KU Leuven. One female founder with the expected 428-bp deletion was selected and crossed with a *Rag2*^*tm1.1Flv*^; *Csf1*^*tm1(CSF1)Flv*^; *Il2rg*^*tm1.1Flv*^; *App*^*tm3.1Tcs*^ male and the progeny were interbred to obtain a *Rag2*^*tm1.1Flv*^; *Csf1*^*tm1(CSF1)Flv*^; *Il2rg*^*tm1.1Flv*^; *App*^*tm3.1Tcs*^; *Csf1R*^*em1Bdes*^. For maintaining the colony, *App*^*NL-G-F*^ *Csf1r*^*ΔFIRE/ΔFIRE*^ males were crossed with *App*^*NL-G-F*^ *Csf1r*^*ΔFIRE/WT*^ females, as five times homozygous females tend to take less care of their progeny. For grafting, *App*^*NL-G-F*^ *Csf1r*^*ΔFIRE/ΔFIRE*^ pups were fostered to CD1 mothers to facilitate their survival.

An extra cohort of *Rag2*^*tm1.1Flv*^; *Csf1*^*tm1(CSF1)Flv*^; *Il2rg*^*tm1.1Flv*^; *App*^*tm3.1Tcs*^ mice (*App*^*NL-G-F*^ mice; mixed C57BL/6, Balb/c background; generated at KU Leuven as previously described in ref. ^16^) was xenotransplanted with human microglia^16^, treated between 6 and 8 months of age and used for the in vivo phagocytosis assay. To establish proper gating for this experiment, we also included *Rag2*^*tm1.1Flv*^; *Csf1*^*tm1(CSF1)Flv*^; *Il2rg*^*tm1.1Flv*^; *App*^*em1Bd*es^ mice (*App*^*Hu*^ *mice;* mixed C57BL/6, Balb/c background; generated at KU Leuven as previously described in ref. ^16^) as negative controls. These mice have humanized Aβ sequence^42^ and do not develop Aβ pathology. Like *App*^*NL-G-F*^ mice, they were xenotransplanted with human microglia. Because neither *App*^*Hu*^ nor *App*^*NL-G-F*^ mice are genetically devoid of endogenous mouse microglia, these cells were depleted before transplantation by inhibiting the CSF1 receptor using BLZ945 (Asclepia Outsourcing Solutions, BLZ945) at a dose of 200 mg kg^−1^ on postnatal days 2 and 3 (P2 and P3), as previously described in ref. ^16^.

### Study design

To unravel microglial contributions to anti-Aβ immunotherapy, we used several experimental approaches, summarized below. To calculate the number of mice needed to analyze the impact of treatment on amyloid pathology using immunofluorescence and MSD ELISA, we conducted a priori power analyses using G*Power (sample sizes noted throughout the test). The expected effect size was based on previous publications^1,6,17^. The *α* level and power were set at 5% and 80%, respectively, and a *t*-test was used for the sample size calculation. No statistical methods were used to predetermine sample sizes for the scRNA-seq analysis, but our sample sizes were estimated based on the previous publications^16,41,43^. Mice were randomly assigned to conditions and conditions were randomized to account for potential ordering effects. To avoid litter bias in the mouse experiments, experimental groups were composed of animals from different litters randomly distributed. All analyses were conducted blindly to the experimental condition, and we used an automated GA3 recipe in the NIS-Elements AR software for image analysis.

We excluded one mouse from the scRNA-seq datasets due to extremely low cell counts (likely due to technical errors). For ELISA data, statistical outliers (caused by technical errors) were identified using the ROUT test in Prism 10 (*Q* = 1%) and excluded from further analysis. No additional mice were excluded from the study.

1. To analyze the distribution of the human antibodies in the brain parenchyma, a cohort of *App*^*NL-G-F*^ *Csf1r*^*ΔFIRE/ΔFIRE*^ mice xenotransplanted with human microglia was treated with either Lecanemab or Lecanemab LALA-PG (10 mg kg^−1^) for 2 or 8 weeks (2-week cohort: *n* = 3, one male and two females; 8-week cohort: *n* = 4, two males and two females). One day after the final dose, mice were terminally anesthetized with an overdose of sodium pentobarbital (Vetoquinol, BE-V171692) and were transcardially perfused with ice-cold DPBS supplemented with 5 U heparin (LEO). One hemisphere was snap-frozen for MSD ELISA, while the other was embedded in cold tissue freezing media (Leica, 14020108926) and snap-frozen in 2-methylbutane (Merck, 1.06056) chilled with liquid nitrogen. All samples were stored at −80 °C. For the Nova-ST experiment, two samples from the 8-week cohort per treatment condition (one male and one female) were further processed.
2. The ability of Lecanemab to reduce AD pathology and modulate the microglial transcriptome and phenotype was assessed after the chronic treatment of 4-month-old male and female *App*^*NL-G-F*^ *Csf1r*^*ΔFIRE/ΔFIRE*^ mice dosed weekly intraperitoneally for 8 weeks with 10 mg kg^−1^ Lecanemab (*n* = 21, 12 males and 9 females), Lecanemab LALA-PG (*n* = 11, 6 males and 5 females) or human IgG1 control (*n* = 17, 6 males and 11 females). One day after the final dose, mice were terminally anesthetized with an overdose of sodium pentobarbital and transcardially perfused with ice-cold DPBS supplemented with 5 U of heparin. The brain was quickly removed and halved along the medio-sagittal line. The right hemisphere was fixed by immersion in 4% formaldehyde (Sigma-Aldrich, 1.00496) for 24 h, followed by storage in DPBS containing 0.01% sodium azide (NaN_3_; Sigma-Aldrich, S8032) until cut. The left hemisphere was either (1) snap-frozen for MSD ELISA, or (2) immediately processed for human microglia isolation. This treatment paradigm was repeated four times using independent batches of xenotransplanted mice (derived from independent differentiations) and different antibody batches. All replication attempts were successful.
3. To demonstrate that microglia are required for the efficacy of Lecanemab, a cohort of *App*^*NL-G-F*^ *Csf1r*^*ΔFIRE/ΔFIRE*^ mice nonxenotransplanted with human microglia was treated for 8 weeks with either IgG1 (*n* = 10, six males and four females) or Lecanemab (*n* = 12, six males and six females) and brains were processed as described above. This treatment paradigm was repeated twice using different antibody batches. All replication attempts were successful.
4. To demonstrate that Lecanemab’s effect on plaque load is not hampered by the presence of adaptive immune cells, we repeated the experiments in a cohort of immunocompetent *App*^*NL-G-F*^ mice treated with mAb158 (a mouse version of Lecanemab; *n* = 9, five males and five females), its engineered LALA-PG variant (*n* = 9, five males and five females) and a control mouse IgG2a (*n* = 9, five males and five females). These mice were treated for 8 weeks, and their brains were processed for MSD ELISA as described above.
5. For in vivo phagocytosis, a cohort of xenografted *App*^*NL-G-F*^ mice were treated with either IgG1 (*n* = 8, four males and four females) or Lecanemab (*n* = 7, three males and four females) from 6 to 8 months; xenografted *App*^*Hu*^ mice^42^ lacking amyloid deposition (*n* = 4, two males and two females) served as negative controls for Methoxy‑X04 gating. This experiment was repeated twice using independent batches of xenotransplanted mice (derived from independent differentiations) and different antibody batches.

### Spatial transcriptomics with Nova-ST

#### Tissue collection and permeabilization for Nova-ST with immunofluorescence

We performed Nova‑ST on repurposed NovaSeq S4 flow cells following ref. ^22^ and associated resources^44,45^. Tissue freezing media‑embedded hemispheres were sagittally cryosectioned. RNA quality from initial 50-µm trimming sections (RIN > 8) was verified before analysis. Tissue optimization used the 10x Visium Tissue Optimization kit with methanol fixation and immunofluorescence, according to the manufacturer’s protocol (Visium Spatial Tissue Optimization Reagents kit, User Guide CG000238, Ref F). Permeabilization used pepsin (Sigma-Aldrich, P7000-25G; 0.65 U µl^−1^ in 0.01 N HCl), tested over 3–45 min; 14 min gave optimal RNA footprints. Additional information on tissue collection and optimization of tissue permeabilization can be found in Supplementary Methods.

#### Spatial transcriptomics analysis using Nova-ST with immunohistochemistry

For Nova‑ST runs, 10-µm sections were mounted on Nova‑ST chips, fixed in methanol at −20 °C for 30 min and blocked (2× blocking buffer prepared with 6× SSC buffer (Sigma-Aldrich, S6639-1L), 4% BSA (Sigma-Aldrich, A9576-50ML), 0.2% TX-100 (Sigma-Aldrich, X-100-5ML) and 10% (vol/vol) Ribonucleoside Vanadyl Complex (NEB, S1402S)). Primary stains were goat antihuman IgG Alexa 647 (goat antihuman IgG Alexa Fluor 647 (40 µg ml^−1^; Thermo Fisher Scientific, A-21445)) to visualize the human antibodies, followed by washes and staining with rabbit anti‑Aβ D54D2 Alexa 594, mouse antihuman CD45 Alexa 488, and DAPI (rabbit anti-Aβ (D54D2) Alexa Fluor 594, 80 µg ml^−1^ (Bioké, 35363S); mouse antihuman CD45 Alexa Fluor 488, 80 µg ml^−1^ (BioLegend, 304017); DAPI, 3,4 µg ml^−1^ (Sigma-Aldrich, D9542)). Images were acquired on Nikon NiE 8-staged using ×10/0.45 air objective (Plan Apo 10× Lambda CFI Eclipse DIC N1; Nikon, MRD00100) for the whole chip and Nikon AX Confocal Microscope System using a ×40/0.75 air objective (Plan Apo Lambda S 40×C Sil; Nikon, MRD73400) for high magnification; three-dimensional surface reconstructions were performed in Imaris. Additional information on the image acquisition and analysis can be found in Supplementary Methods. After imaging, coverslips were removed and tissue was permeabilized with pepsin (Sigma-Aldrich, P7000-25G; 0.65 U µl^−1^ in 0.1 N HCl, pH 2.0) for 14 min at 37 °C, and then subjected to reverse transcription on‑chip. A detailed description of the spatial transcriptomics pipeline can be found in Supplementary Methods.

#### Region-of-interest (ROI) detection

Single fluorescence and brightfield images were stitched using Fiji (MultiView-Stitcher) with a 15% overlap. The resulting overlays of immunostainings and deep-sequencing images were imported into QuPath (v0.4.3) to identify cortical regions. Within these regions, we selected ROIs (D54D2^+^ plaque regions) based on a size exclusion criterion of <20 µm^2^. A semi-automated script in QuPath was used to detect plaque ROIs, which were then manually screened to exclude artifacts. Finally, GeoJSON files were exported for subsequent analysis.

#### Nova-ST data alignment, quality control and preprocessing

A combined human (hg38) and mouse (mm10) STAR index was created from the 10x Genomics, Genomics GRCh38-and-mm10-2020-A index using STAR (v2.7.11a). This index was used together with the NovaScope Pipeline (v1.1)^46^, using spatula (v1.0.0) and STAR to preprocess, map and quantify the data using the following two workflows: sge-per-run and transcript-per-unit. Mapped and quantified data were loaded and binned into hexagonal bins (TDs) with a width, and center-to-center distance of 40 μm using custom Python code, images were exported at a resolution of 1px:1μm and used for alignment with immunofluorescence images previously collected. Images were aligned to spatial data using the Landmark Correspondences plugin in Fiji. Multiple fiducial circles, visible in both the 488 IF channel and the spatial data, were selected as landmarks. An affine transformation was then applied to achieve precise alignment.

Expression matrices of all TDs were read into Scanpy (v1.9.8)^47^ and preprocessed. Genes were subset to retain only those mapping to the human transcriptome. Quality control metrics were calculated and TDs with fewer than 30 UMIs and greater than 250 UMIs were removed. GeoJSON files specifying cortical regions and plaque ROIs were read into Python using geopandas (v0.12.0) and overlapped with the spatial object (Extended Data Fig. 2a). TDs falling in the cortical region were retained and the distance from the center of each TD to the edge of the nearest segmented plaque was calculated using Scipy’s (v1.10.1) KDTree package (https://github.com/scipy/scipy/blob/main/scipy/spatial/kdtree.py).

#### Distance-based DE analysis

DE analyses of TDs with respect to distance to pathology were conducted by fitting generalized linear models in the Lecanemab-treated and Lecanemab LALA-PG-treated mice separately. Analysis was performed only on TDs within 200 µm of a plaque edge to limit the biasing effect of outlying TDs, and on genes expressed in at least 90 TDs. Each generalized linear model was tested for DE using EdgeR’s (v3.40.0) quasi-likelihood *F* test (QLFTest), which accounts for uncertainty in dispersion estimation. Multiplicity correction was performed by applying the Benjamini–Hochberg method on the associated *P* values, and a significance threshold of *P*_adj_ < 0.05 was used for all DEs.

#### Immunofluorescence on vibratome sections

A total of 30‑µm sagittal sections were cut with a Leica VT1000S. Target lateral coordinate was ~1.56 mm using Franklin and Paxinos^48^. Sections underwent citrate antigen retrieval, permeabilization in 0.2% Triton X‑100 (Sigma-Aldrich, T9284-100ML), X‑34 staining (Sigma-Aldrich, SML1954) for 20 min and blocking in 5% donkey serum in DPBS‑Triton. Primary antibodies used included antihuman P2RY12, anti‑Aβ N‑terminus 82E1, anti‑IBA1, anti‑LAMP1, anti‑IBA1, anti‑Homer1, anti‑Synaptophysin, anti‑OPN, anti-HLA, anti-CD9 and anti-CD68, followed by Alexa Fluor secondaries. Detailed information on the antibodies used in this study, immunofluorescence protocol, image acquisition and image analysis can be found in Supplementary Methods.

#### Brain extraction, soluble and insoluble Aβ, and MSD quantification

Frozen hemispheres were homogenized in 10× (wt/vol) P‑TER buffer (Thermo Fisher Scientific, 78510) with Complete Protease Inhibitor Cocktail (Roche, 11697498001) and PhosSTOP Phosphatase Inhibitor Cocktail (Roche, 4906845001). After low‑speed clearing, ultracentrifugation pelleted insoluble material. Supernatants were collected as soluble fractions. Pellets were extracted with 6 M guanidine hydrochloride (GuHCl; Sigma-Aldrich, G3272), 50 mM Tris–HCl (Invitrogen, AM9856) and protease inhibitor cocktail (pH 7.6), followed by sonication, incubation at 25 °C, and ultracentrifugation. The resulting supernatant was then diluted 12-fold in GuHCl diluent, composed of 20 mM phosphate buffer—NaH_2_PO_4_·2H_2_O (VWR, 928015.294), Na_2_HPO_4_·2H_2_O (Supelco, 106580.100), 0.4 M NaCl (VWR, 27788.297), 2 mM EDTA (Bioworld, 40120777-1), 10% Block Ace (Bio-Rad, BUF029), 0.2% BSA (Miltenyi, 130-091-376), 0.05% NaN_3_, 0.075% CHAPS (Sigma-Aldrich, C3023) and protease inhibitor cocktail (pH 7.0). MSD plates were coated overnight at 0.5 µg ml^−1^ with capture antibodies specific for Aβ38, Aβ40 or Aβ42 neoepitopes. After blocking, samples were assayed with sulfo‑TAG-labeled anti‑N‑terminal Aβ detection antibody (250 ng ml^−1^, homemade mouse monoclonal against the N-terminal sequence of human Aβ, in collaboration with M.D.). Plates were read on an MSD Sector Imager 2400A and MESO QuickPlex SQ 120MM (for the experiment performed on immunocompetent mice). Recombinant standards were Aβ_1–38_, Aβ_1–40_ and Aβ_1–42_. Additional information can be found in Supplementary Methods.

#### Ex vivo plaque clearance assay

Human microglia‑like cells were differentiated from day 25 or day 32 precursors in microglia differentiation medium based on the details discussed in ref. ^49^. Cryosections (10 µm) from immunocompetent 6‑month *App*^*NL‑G‑F*^ brains were pre-incubated with 10 µg ml⁻¹ Lecanemab, Lecanemab LALA‑PG or IgG1 for 1 h at 37 °C. Human microglia were seeded at 5 × 10⁵ cells per well and incubated for 72 h at 37 °C, 5% CO_2_. For OPN titrations, cells were stimulated with 0–1,350 ng ml⁻¹ human OPN (Preprotec, 120-35) under identical conditions. Sections with or without cells were fixed and stained for Aβ (82E1) and human microglia (CD9). Whole‑section images were acquired on Nikon AX confocal (×10 with ×1.6 electronic magnification). Thus, 82E1‑positive area was quantified by NIS‑elements GA3 and normalized to section area. Additional information on the in vitro plaque clearance assay, image acquisition and analysis can be found in Supplementary Methods.

#### Isolation of human microglia for flow cytometry and scRNA‑seq

Human microglia were isolated from the mouse brain as previously described^16^. After heparinized DPBS perfusion, one hemisphere was immediately processed in fluorescence-activated cell sorting buffer (DPBS, 2-mM EDTA and 2% FBS; Gibco, 10270106) as well as actinomycin‑D (5 µM; Sigma-Aldrich, A1410-5MG) to minimize ex vivo activation^50^. Brains were dissociated with the Miltenyi Neural Tissue Dissociation Kit P (Miltenyi, 130-092-628) as well as actinomycin‑D. Myelin was removed by 30% isotonic Percoll (GE Healthcare, 17-5445-02). Fc receptors were blocked (mouse, 0.1 mg ml^−1^ (Miltenyi, 130-092-575) and human, 0.1 mg ml^−1^ (Miltenyi, 130-059-901)). Cells were stained with PE-Pan-CD11b (20 µg ml^−1^; Miltenyi, 130-113-806), BV421-mCD45 (2 µg ml^−1^; BD Biosciences, 563890), APC-hCD45 (20 µg ml^−1^; BD Biosciences, 555485), Total-Seq A cell hashing antibodies (2 µg ml^−1^; BioLegend) and viability dye (0.5 µg ml^−1^, eFluor 780; Thermo Fisher Scientific, 65-0865-14). Sorting was performed at 4 °C on a MACSQuant Tyto (Miltenyi), gating on CD11b and human CD45. For in vivo phagocytosis, Methoxy‑X04 (10 mg kg^−1^, intraperitoneal) was administered 3 h before euthanasia. After isolation, cells were stained for CD11b, mouse CD45, human CD45 and viability, fixed and permeabilized, then stained intracellularly for CD68. Acquisition was performed using a BD Fortessa. Methoxy‑X04 gating was set using xenografted *App*^*Hu*^ mice as negative controls. Data were analyzed in FlowJo (v10.8.1). Additional information on the procedure used to isolate the microglia and perform the in vivo phagocytosis assay can be found in Supplementary Methods.

For scRNA-seq, 15,000–20,000 human microglia (CD11b^+^, hCD45^+^) from each mouse were sorted on the MACSQuant Tyto and diluted to a final concentration of 1,000 cells per µl. Because all the samples were individually hashed using Total-Seq A cell hashing antibodies (2 µg ml^−1^; BioLegend), 2,000–4,000 human microglia per animal were pooled and loaded onto the Chromium Next GEM Chip G (PN 2000177). The DNA library preparations were generated according to the manufacturer’s instructions (CG000204 Chromium Next GEM Single Cell 3′ Reagent Kits v3.1). In parallel, the hashtag oligo (HTO) libraries were prepared according to the manufacturer’s instructions (BioLegend, Total-Seq A Antibodies and Cell Hashing with 10x Single Cell 3′ Reagent Kit v3 3.1 protocol) using 16 cycles for the index PCR. A total of five libraries containing 12 biological replicates were sequenced (GAL002, GAL004, GAL005, H1 and H2; Extended Data Fig. 4a,b), targeting a 90% messenger RNA and 10% hashtag oligo library (50,000 reads per cell), on a HiSeq4000 (Illumina) platform with the recommended read lengths by the 10x Genomics workflow.

### Single-cell sequencing data analysis

#### Alignment, preprocessing and quality control

The 10x Genomics Cell Ranger software (v6.1.2) was used to align reads to a combined human/mouse reference genome (GRCh38 and mm10), demultiplex cellular barcodes and quantify UMIs and HTO. The UMI and HTO count matrices were loaded into the Seurat R package (v4.1.1)^51^. Genes whose transcripts were identified in <3 cells, as well as cells with <100 unique transcripts, were removed from the expression matrices. Demultiplexing of cells to their original samples was performed as described in ref. ^52^ and in Supplementary Methods. Additional quality control removed cells by 2.5 median absolute deviations on log1p UMI count, log1p genes and mitochondrial percentage per library, discarded cells with >5% mouse‑aligned reads, and removed intrasample doublets detected by DoubletFinder^53^ (v2.0.3). The final dataset contained 22,841 high‑quality human microglia.

#### Normalization, integration and clustering

Gene counts from each of the five libraries were individually normalized using ‘SCTransform()’^54^. The ‘SelectIntegrationFeatures()’ function was used to identify the 3,000 most variable features in each library. The ‘PrepSCTIntegration()’, ‘FindIntegrationAnchors()’ and the ‘IntegrateData()’ functions were used to integrate the libraries. A principal component analysis was performed on the integrated expression matrix, after which uniform manifold approximation and projection (UMAP)^55^ embeddings were created with ‘RunUMAP()’, using the first 30 principal components as input. A shared nearest neighbor graph was constructed using ‘FindNeighbors()’. Unbiased clustering by the Louvain algorithm (‘FindClusters()’) with a resolution of 0.35 identified 11 clusters (Extended Data Fig. 4b,c). The ‘PrepSCTFindMarkers()’ and ‘FindAllMarkers()’ functions were run to find marker genes for each cluster. Cluster 10 was identified as the macrophage cluster and removed from the analysis (Extended Data Fig. 4c,d). The above-described steps were repeated for the remaining microglial cells (*n* = 22,420) using the same parameters, except for the unbiased Louvain clustering, which was run with a resolution of 0.15 and identified nine clusters.

#### Single-cell differential gene expression analysis

DEs between clusters and treatment conditions in the single-cell analysis were performed on SCT-normalized counts using ‘PrepSCTFindMarkers()’ and ‘FindMarkers()’. *P* values were calculated using the Wilcoxon rank-sum test and corrected for multiple testing using the Bonferroni method. GSEA^56^ was performed using the clusterProfiler^57^ package (v4.6.2), ranking genes on their log_2_(FC). Specific functions and parameters used are outlined in Supplementary Methods.

#### Differential abundance assessment of microglial cell states

To test for differential abundance of cell states in association with Lecanemab treatment, we used mixed-effects modeling association of single cells^58^ (v0.1.0, R; Extended Data Fig. 4c). Treatment status was used as a fixed effect and mouse ID as a random effect.

#### WGCNA

High-definition WGCNA (hdWGCNA)^59^ (v0.3.0, R) was used to perform coexpression network analysis. First, we filtered out genes expressed in less than 5% of all cells. We then generated metacells for each sample, aiming for 200 per sample. A soft power was chosen to achieve a scale‑free topology fit ≥0.9 with a signed‑hybrid coexpression network. Specific functions and parameters used are specified in Supplementary Information. Modules and eigengenes were computed and the top ten hub genes were defined as genes with the top kME per module. Overrepresentation of GO^60,61^ terms in the modules was assessed with clusterProfiler’s ‘enrichGO()’ function (Fig. 4e and Supplementary Table 2), with the following parameters: minGSSize = 10, maxGSSize = 500, pvalueCutoff = 0.05, qvalueCutoff = 0.2, pAdjustMethod = ‘BH’*.*

### Statistical analysis and reproducibility

Statistical analyses and data visualization were performed using GraphPad Prism (v9) and R (v4.2.3). Data are presented as scatter dot plot with bar, and the line at the mean ± s.e.m. (s.e. of the mean). All *n* values represent individual animals, unless stated otherwise (that is, ex vivo plaque clearance assay, where each *n* represents an independent experiment). When appropriate, animals were randomly assigned to conditions and conditions were randomized to account for potential ordering effects. To avoid litter bias in the mouse experiments, experimental groups were composed of animals from different litters randomly distributed. All analyses were conducted either blindly to the experimental condition or using an automated GA3 recipe in NIS-Elements AR software. For all the ELISA data, statistical outliers (caused by technical errors) were identified using the ROUT test in Prism 10 (*Q* = 1%) and excluded from further analysis. Normality of residuals was assessed using the Shapiro–Wilk test, and the data met the assumptions of the statistical tests used. Comparisons between two groups following a normal distribution were analyzed using two-tailed unpaired *t*-test, while comparisons between two groups not following a normal distribution were analyzed using the Mann–Whitney test. When three groups were compared and data were normally distributed, ordinary one-way analysis of variance was used; if significant, it was followed by Bonferroni’s multiple comparisons test. When data were not normally distributed, ranks were compared with the Kruskal–Wallis test followed by Dunn’s multiple comparisons test. To determine the statistical significance of the difference between microglia and no-microglia curves upon OPN stimulation, we used a modified chi-squared method (as described in ref. ^62^). Notably, this method includes a correction for the deviation from normality. To determine the overall statistical significance of the differences in plaque area distribution, we used the Anderson–Darling test. For pairwise comparisons between distributions, we performed the Kolmogorov–Smirnov test. To account for multiple comparisons (three in total), we applied a Bonferroni correction by multiplying the obtained *P* values by three. The statistical tests are reported in the figure legends and for all analyses, *α* = 0.05. A detailed description of statistical analysis and the number of mice used in this study is reported in Supplementary Table 6.

### Reporting summary

Further information on research design is available in the Nature Portfolio Reporting Summary linked to this article.

## Online content

Any methods, additional references, Nature Portfolio reporting summaries, source data, extended data, supplementary information, acknowledgements, peer review information; details of author contributions and competing interests; and statements of data and code availability are available at 10.1038/s41593-025-02125-8.

## Supplementary information

Supplementary Information Supplementary Methods.

Reporting Summary

Supplementary Table 1 Human microglial transcriptomic modules identified by WGCNA. Modules were derived using hdWGCNA on scRNA-seq data from microglia isolated from Lecanemab-treated and IgG1-treated mice.

Supplementary Table 2 GO enrichment analyses of the human microglial transcriptomic modules identified by WGCNA (top 20 terms). Enrichments were assessed by a one-sided hypergeometric overlap test. *P* values were adjusted using the BH correction.

Supplementary Table 3 Overlap between the pink module (identified by WGCNA) and microglial cell state^16,26,27^ marker genes. Enrichments between genes in the pink module and predefined microglial cell state marker gene sets (rows) were assessed by a one-sided hypergeometric overlap. For each module–cell state pair, the table reports the BH-adjusted *P* value (*P*_adj_) and the genes shared between the module and the cell state (OverlapGenes).

Supplementary Table 4 DE of FCGRs in the human microglia. DE analyses were performed using a two-sided Wilcoxon rank-sum test. *P* values were adjusted with Bonferroni correction.

Supplementary Table 5 Summary of antibody sequences used in this study.

Supplementary Table 6 Detailed description of the statistical analysis used in this study.

Supplementary Video 1 Three-dimensional surface reconstruction of Lecanemab localization in microglia. This video presents a 3D surface reconstruction of D54D2-positive plaques in a Lecanemab-treated mouse, generated using a surface reconstruction. The anti-IgG signal (labeling Lecanemab) is detected within human CD45^+^ microglia, highlighting its localization inside microglial surfaces. This reconstruction corresponds to Fig. 1a (top panel).

## Source data

Source Data Fig. 1 Statistical source data.

Source Data Fig. 2 Statistical source data.

Source Data Fig. 3 Statistical source data.

Source Data Fig. 4 Statistical source data.

Source Data Fig. 5 Statistical source data.

Source Data Extended Data Fig. 2 Statistical source data.

Source Data Extended Data Fig. 3 Statistical source data.

## Data Availability

The Nova-ST and scRNA-seq data generated in this study have been deposited at the Gene Expression Omnibus (GEO) database and are publicly available as of the date of publication. GEO accessions are listed as follows: Nova-ST (GSE297667) and scRNA-seq data (GSE297665). Source data are provided with this paper. Other data are available upon request. Source data are provided with this paper.

## Code availability

The source code used in this study is available via GitHub at https://github.com/mmzielonka/AlbertiniZielonka_Lecanemab_2025.git and Zenodo at 10.5281/zenodo.17098381 (ref. ^63^).
