# Supplementary material for: The Alzheimer’s therapeutic Lecanemab attenuates Aβ pathology by inducing an amyloid-clearing program in microglia
Source: Nat Neurosci. 2025 Nov 24;29(1):100–10. doi: 10.1038/s41593-025-02125-8 (PMC12779577; doi:10.1038/s41593-025-02125-8)
Supplement: Supplementary file 1 — Supplementary Methods. [file 41593_2025_2125_MOESM1_ESM.pdf]

nature neuroscience

Supplementary information

https://doi.org/10.1038/s41593-025-02125-8

The Alzheimer's therapeutic Lecanemab attenuates Aβ pathology by inducing an amyloid-clearing program in microglia

In the format provided by the authors and unedited

Supplementary Methods

Spatial Transcriptomics with Nova-ST

Tissue collection for Nova-ST with immunofluorescence

Tissue collection was performed as described in[1]. In brief, tissue freezing media-embedded hemispheres (Leica, #14020108926) were cryosectioned sagittally using a CryoStar NX70 cryostat (Thermo Fisher Scientific). First, 5–10 serial sections of 50 µm thickness were cryo-sectioned from a region farther away from the region of interest. These serial sectioned tissue freezing media scrolls were put into a 2 mL lo-bind tube (Eppendorf, #0030108078) and stored at -80°C. The tissue scrolls were washed with 1 mL of ice-cold PBS at 4°C. Total RNA extraction from the spun-out tissue was performed using innuPREP mini-RNA kit (Analytik Jen, #845-KS-2040250). Manufacturer recommendations were followed to extract total RNA. Elution was performed in 30 mL of nuclease free water, NFW (Thermo Fisher Scientific, #10977035). The quality of the total RNA was assessed using Pico RNA kit (Agilent) and was above 8 for all samples.

Optimization of tissue permeabilization for Nova-ST with immunofluorescence

The tissue optimization for Nova-ST was performed using a 10X Genomics Visium Spatial Tissue Optimization kit, according to the manufacturer's protocol (Visium Spatial Tissue Optimization Reagents kit, User Guide CG000238.Ref F). Briefly, 8x 10 µm tissue sections are placed on the 8x available slots on the Visium Spatial Tissue Optimization Slide (PN: 300394). Methanol fixation, immunofluorescence staining and Imaging was performed as described in the 10X Genomics demonstrated protocol (CG000312.RevF). The permeabilization test is performed as per manufacturer's recommendations, except the permeabilization enzyme (PN: 2000214) was replaced by 0.65U μl^{-1} working stock of Pepsin enzyme in 0.01 N HCl (pH 2.0) (Sigma-Aldrich, P7000-25G). The optimal permeabilization time was determined by performing the permeabilization time series of eight different time points ranging from 3 mins – 45 mins, and used for the downstream spatial transcriptomics workflow[2] (further details in[1]). Similar to other spatial techniques such as 10X Genomics Visium & BGI STomics, the optimal permeabilization time was assessed based on the strongest fluorescence signal with the lowest signal diffusion (crispness of the RNA footprint). Based on our assessment, we found the most optimal permeabilization time for the mouse brain section to be 14 mins.

Spatial Transcriptomics analysis using Nova-ST

The Nova-ST spatial transcriptomics analysis was performed using Nova-ST chips prepared from a repurposed NovaSeq 6000 S4 flow cell as described in the publication[2] and resources[3]. The spatial transcriptomics workflow was followed as per the publication[2] (additional details in[1]). Briefly, the Nova-ST chip was removed from the storage buffer and washed with nuclease free water and dried at 37°C. Next, a 10 µm tissue section from the desired region of interest (lateral 1.56 mm) was prepared from the tissue cryo-block and placed on the Nova-ST chip and thawed to attach the tissue layer on the surface of the chip. The chip was then stored at -80°C until the spatial transcriptomics experiment. For

performing the ST assay, the tissue was dried on a 37°C hot plate for 2 mins and the chip was then dipped into 100% methanol (Sigma-Aldrich, #34860) at -20°C and incubated for 30 mins to fix and permeabilize the tissue. After the methanol fixation, the Nova-ST chip was blocked using 200 µl of blocking buffer (2X blocking buffer prepared with 6X SSC buffer, Sigma-Aldrich #S6639-1L; 4% BSA, Sigma-Aldrich #A9576-50ML; 0.2% TX-100, Sigma-Aldrich #X-100-5ML and 10% (v/v) Ribonucleoside Vanadyl Complex, NEB, #S1402S) for 5 minutes. After removing the blocking buffer, we added 110 µl of conjugated anti-IgG primary antibody staining mix (2X blocking buffer; goat anti-Human IgG- Alexa Fluor™ 647: 40 µg ml^{-1}, Thermo Fisher Scientific, #A-21445; 6U µl^{-1} of RNAse inhibitor, NEB, #M0314L) and incubated for 30 mins. After the incubation, the chip was washed 5x times with wash buffer (2X blocking buffer & 10% (v/v) Ribonucleoside Vanadyl Complex, NEB, #S1402S). After the last wash with the buffer, we added 110 µl of conjugated anti-hCD45 and anti-D54D2 antibodies staining mix, together with DAPI (2X blocking buffer; rabbit anti-β-Amyloid (D54D2)- Alexa Fluor™ 594: 80 µg ml^{-1}, Bioké, #35363S; mouse anti-Human CD45-Alexa Fluor™ 488: 80 µg ml^{-1}, BioLegend, #304017; DAPI: 3,4 µg mL^{-1}, Sigma-Aldrich, #D9542; 6U µl^{-1} of RNAse inhibitor, NEB, #M0314L) and incubated for 30 mins. After the incubation, the chip was washed 5x times with wash buffer (2X blocking buffer & 10% (v/v) Ribonucleoside Vanadyl Complex, NEB, #S1402S). The chip was then washed by dipping 20X times in 3X SSC buffer. 20 µl of glycerol mounting medium (85% glycerol, Sigma-Aldrich, #G5516-100ML and 2U µl^{-1} of RNAse inhibitor, NEB, #M0314L) was added onto the tissue on the chip and after placing a coverslip, the tissue on the Nova-ST chip was imaged for the DAPI and antibody stains. For downstream tissue alignment and analysis of the spatial transcriptomics data, images were registered for using Nikon NiE 8-staged driven by NIS-Elements AR (6.10.01) software, using a 10x/0.45 air objective (Plan Apo 10x Lambda CFI Eclipse DIC N1, Nikon, #MRD00100). Images had a resolution of 2048 x 2048, and 0.63 µm pixel size. Additional high magnification images were taken with Nikon AX Confocal Microscope System driven by NIS-Elements AR (5.41.01) software, using a 40x/0.75 air objective (Plan Apo Lambda S 40XC Sil, Nikon, #MRD73400). Ten stacks were acquired using a 0.8 µm z-interval and images had a resolution of 2048 x 2048, and 0.12 µm pixel size. 3D surface reconstructions were performed with Imaris Software (Bitplane).

After imaging, the coverslip was removed from the chip and the chip washed with excess of 3X SSC buffer. After incubating the chip in 3X SSC for 3 minutes, the chip was subject to tissue permeabilization with 120 µl of tissue permeabilization mix of 0.65U µl^{-1} working stock of Pepsin enzyme in 0.1N HCl (pH 2.0) (Sigma-Aldrich, #P7000-25G) at 37°C. The optimal permeabilization time estimated from the tissue permeabilization analysis (14 mins) was used for the transcriptomics analysis. After washing the chip with successive rounds of 0.1X SSC wash buffer and 1X RT wash buffer, the reverse transcription mix (1.05 U µl^{-1} RNAse Inhibitor, Lucigen, #30281-1; 4.5% Ficoll PM-400, Sigma-Aldrich, #F5415-50 ML; 1.05 mM dNTPs 25 mM, Thermo Fisher Scientific, #R1121; 10 U µl^{-1} Maxima h-RT, Thermo Fisher Scientific, #EP0753) was added to the chip and incubated at 42°C overnight (18-24 hrs) to complete the reverse transcription. The chip was then subjected to successive rounds of wash with 0.1X SSC and 1X Exo I buffer. Chip was then subjected to exonuclease digestion (Exonuclease I, NEB #M0293L) at 37°C for 45 minutes followed by

tissue removal digestion (100 mM Tris pH 8.0, Thermo Fisher Scientific, #J22638.AE; 200 mM NaCl, Thermo Fisher Scientific, #AM9760G; 2% SDS, Thermo Fisher Scientific, #AM9822; 5 mM EDTA, Thermo Fisher Scientific, #AM9260G and 16 mU μl^{-1} of Proteinase K, NEB #P8107S) to remove the leftover tissue from the Nova-ST chip by incubating the chip in the tissue removal mix at 37°C for 60 minutes. Caustic denaturation (0.1 N NaOH pH 13, Sigma-Aldrich, #72068-100ML) was performed on the chip to remove the RNA strands from the first strand product on the Nova-ST chip. After the 3 rounds of neutralization washes (100 mM Tris pH 7.5, Thermo Fisher Scientific, #15567027) and nuclease free water washes, the chip was then subjected to second strand synthesis reaction. In this reaction the chip was submerged into second strand synthesis mix (1X NEB buffer 2, NEB, #M0212L; 11 uM RPE randomer, IDT technologies; 0.55 U μl^{-1} of Klenow exo (-) fragment, NEB, #M0212L) and incubated for 120 mins. The second strand cDNA product was extracted from the Nova-ST chip using caustic denaturation (0.1 N NaOH pH 13, Sigma-Aldrich, #72068-100ML). The denatured product was neutralized with 100 mM Tris pH 7.0, (Thermo Fisher Scientific, #AM9850G) and subsequently purified with 1.8X Ampure XP beads (Beckman Coulter, #A63882) using the manufacturer's recommendation. The purified library was PCR amplified and purified as described in the protocol. After the quality assessment using a bioanalyzer (Agilent) and Qubit (Thermo Fisher Scientific), library normalization was performed. Sequencing library preparation was performed using indexed PCR amplification of the library. Library purification was performed with 2X successive rounds of Ampure XP purification (0.8X followed by 1.0X purification). The rest of the purification protocol was performed as per manufacturer's recommendations.

Nova-ST libraries were sequenced with NextSeq 2000 (Illumina) for shallow sequencing (for quality estimation of the sample) and MGISEQ-2000 (BGI) sequencing platforms for deeper sequencing. Sequencing of the Nova-ST libraries were performed with paired end sequencing of 34 bps (read 1), 85 bps (read 2), 8 bps (index 1) and 8 bps (index 2). MGISEQ-2000 sequencing was done at the MGI Hong Kong SAR sequencing facility. Before being able to sequence on the MGI platforms, the Nova-ST libraries needed to undergo a conversion step using MGIEasy Universal DNA Library Prep kit. Briefly, the sequencing libraires were circularized using the splint-ligation step and the circularized libraries were converted to single-stranded DNA copies. DNA nanoballs were prepared from the circularized ssDNA using Rolling Circle Amplification (RCA). DNB libraries generated were then flown through the patterned flow cell of the MGISEQ-2000RS High-Throughput Sequencing kit. For a targeted sequencing saturation of 50-60%, sequencing were performed to a depth of 1 billion reads per sample.

Visualization of gene set expression levels in TDs

To visualize the expression level of gene sets in TDs, TD expression levels were first normalized using Scanpy's *sc.pp.normalize_total()* and *sc.pp.log1p()* functions. Relative expression levels of gene sets were determined using the *sc.tl.score_genes()* function, and *sc.pl.spatial()* was used to spatially visualize the resultant scores overlaid over the plaque segmentation image (**Fig. 5c**, **Extended Data Fig. 5d-f**).

Immunofluorescence on vibratome sections

Thirty µm-thick sagittal slices were cut using a vibratome (Leica, #VT1000S). At least 2 brain slices per mouse covering the regions of interest (lateral 1.56 mm) were selected following the mouse brain atlas of Franklin and Paxinos[4]. After 3 washes in PBS, antigen retrieval was performed by microwave boiling in 10 mM tri-sodium citrate buffer pH 6.0. Slices were then permeabilized in PBS containing 0.2% Triton X-100 for 15 minutes. After permeabilization, slices were stained with X-34 staining solution (10 µM X-34 (Sigma-Aldrich), 20 mM NaOH (Sigma-Aldrich), and 40% ethanol in PBS) for 20 min at room temperature. Slices were washed three times with 40% ethanol in PBS for 2 min and twice with PBS + 0.2% Triton for 5 min. Afterwards, slices were blocked with PBS-T with 5% donkey serum at room temperature for 1h and stained with primary antibodies overnight at 4 °C with gentle agitation (for synaptic staining, anti-Synaptophysin and anti-Homer1 primary antibodies were incubated for 2 consecutive nights). Primary antibodies, rabbit anti-human P2RY12 (0.2 µg ml^{-1}; Atlas Antibodies, #HPA013796), mouse anti-amyloid beta (N) (clone 82E1) antibody (0.2 µg ml^{-1}; IBL, #10323), guinea pig anti-IBA1 antibody (2 µg ml^{-1}; Synaptic systems, #234308), rat anti-LAMP1 (4 µg ml^{-1}; DSHB, #1D4B-c), rabbit anti-IBA1 antibody (1 µg ml^{-1}; Wako, #019-19741), rabbit anti-Homer1 antibody (2 µg ml^{-1}; Synaptic systems, #160003), mouse anti-Synaptophysin antibody (2 µg ml^{-1}; Synaptic systems, #101011), goat anti-osteopontin/OPN (2 µg ml^{-1}; R&D System, #AF808), were diluted in PBS-T with 5% donkey serum. The next day, after rinsing three times with PBS-T, secondary fluorochrome-conjugated antibodies were added to PBS-T with 5% donkey serum for 2 h at room temperature. The following secondary antibodies were used: goat anti-rabbit, Alexa Fluor™ 647 (2 µg ml^{-1}; Invitrogen, #A21245); goat anti-mouse, Alexa Fluor™ 647 (2 µg ml^{-1}; Invitrogen, #A21236); goat anti-guinea pig, Alexa Fluor™ 488 (2 µg ml^{-1}; Jackson Immunolab, #106-545-003); goat anti-rat, Alexa Fluor™ 594 (2 µg ml^{-1}; Invitrogen, #A11007); donkey anti-mouse, Alexa Fluor™ 647 (2 µg ml^{-1}; Invitrogen, #A31571); donkey anti-rabbit, Alexa Fluor™ 594 (2 µg ml^{-1}; Invitrogen, #A21207); goat anti-mouse, Alexa Fluor™ 546 (2 µg ml^{-1}; Invitrogen, #A21123); donkey anti-goat, Alexa Fluor™ 488 (2 µg ml^{-1}; Invitrogen, #A11055). After rinsing three times with PBS-T and once with PBS, sections were mounted onto glass slides using the Glycergel mounting media (Agilent, # C0563 and allowed to dry at room temperature.

For showing the colocalization of HLA, CD9 and CD68, after X34 staining, wash and blocking, slices were incubated overnight at 4 °C with gentle agitation with mouse anti-human HLA antibody (5 µg ml^{-1}; Abcam, #ab7856). The next day, after rinsing three times with PBS-T, goat anti-mouse, Alexa Fluor™ 647 (2 µg ml^{-1}; Invitrogen, #A21236) was added to PBS-T with 5% donkey serum for 2 h at room temperature. Sections were washed, blocked and incubated with the following primaries overnight at 4 °C with gentle agitation: mouse anti-human CD9 biotinilated (10 µg ml^{-1}; Biolegend, #312112) and rabbit anti-human CD68 (4 µg ml^{-1}; Abcam, #ab213363). The next day, after rinsing three times with PBS-T, secondary fluorochrome-conjugated antibodies were added to PBS-T with 5% donkey serum for 2 h at room temperature. The following secondary antibodies were used: donkey anti-rabbit, Alexa Fluor™ 594 (2 µg ml^{-1}; Invitrogen #A21207); streptavidin, Alexa Fluor™ 488 (2 µg ml^{-1}; Invitrogen, #A11055). After rinsing three times with PBS-T and once with PBS, sections were mounted onto glass slides using the Glycergel mounting media and allowed to dry at room temperature.

Image acquisition and analysis

For the representative image of P2RY12, IBA1 and X34 expression at 4 months (Extended Data **Fig. 1a**), a large field image of the whole sagittal slice was obtained using a Nikon AX Confocal Microscope System driven by NIS-Elements AR (5.41.01) software, using a 4x/0.2 air objective (Nikon CFI Plan Apochromat λ, Nikon # MRD00045). Tiled images were stitched together with an overlap of 15%. For excitation, 405 nm, 488 nm, and 640 nm laser lines were used. Resonant scanning mode was used with 16x line averaging, and minimal crosstalk was set between the channels. Three stacks were acquired using a 5 µm z-interval and images had a resolution of 2048 x 2046, and 1.07 µm pixel size. Higher magnification representative images (**Extended Data Fig. 1b**) were acquired using a 20x/0.75 air objective (Plan Apo VC 20x DIC N2, Nikon, #MRD00201). Twenty-two stacks were acquired using a 0.757 µm z-interval and images had a resolution of 2048 x 2046, and 0.11 µm pixel size.

For the representative image of CD9, HLA-DR, CD68 and X34 expression at 6 months (**Extended Data Fig. 1c**), images were acquired using an inverted Zeiss LSM 880 microscope with Airyscan detector. The system was equipped with a 20x 1.4 NA Plan-Apochromat objective lens and operated using Zen Black (version 2.3, Carl Zeiss Microscopy GmbH) (0.06 µm pixel size). Ten stacks were acquired using a 1 µm z-interval. Airyscan-acquired images were processed using the default values.

For imaging and analysis of X34, 82E1, LAMP1 and IBA1 after treatment, large field images of the whole slice were obtained using a Nikon AX Confocal Microscope System driven by NIS-Elements AR (5.41.01) software, using a 20x/0.75 air objective (Plan Apo VC 20x DIC N2, Nikon, #MRD00201). Tiled images were stitched together with an overlap of 15%. For excitation, 405 nm, 488 nm, 561 nm, and 640 nm laser lines were used and images were acquired using the same acquisition parameters. Three stacks were acquired using a 2.5 µm z-interval. Resonant scanning mode was used with 16x line averaging, and minimal crosstalk was set between the channels. All the quantification results were generated using the measurement of the whole sagittal section. For each experiment, at least 2 sections per mouse were analyzed. For quantification X34+ and 82E1+ areas (1024 x 1024, 0.86 µm pixel size), we used a semi-automated script in Qupath (v0.4.3)[5]. Briefly, after defining the area of interest (total brain section) with the wand tool, X34+ and 82E1+ areas were identified with the threshold function, and then divided by the total brain section's area. Intensity thresholds were selected manually and used for all the sections, setting the minimum detectable size at 1 μm^2 for X34 and 5 μm^2 for 82E1. The distribution of $X34^+$ plaques based on area was assessed on high-magnification z-stacks (six cortical images per mouse). Thirty-one stacks were acquired using a Nikon AX Confocal Microscope System controlled by NIS-Elements AR (v5.41.01) software, with a 40×/1.25 oil objective (Plan Apo Lambda S 40XC Sil, Nikon, #MRD73400) at a resolution of 2048 × 2048 pixels and a pixel size of 0.21 µm. The area of X34+ plaques was analyzed using an automated GA3 recipe in NIS-Elements AR (5.42.05) software. A total of 975, 731, and 942 plaques were analyzed for IgG1, Lecanemab, and Lecanemab LALA-PG, respectively. Plaque distribution was analyzed using a frequency distribution approach, with plaque sizes grouped into intervals of 20 µm to facilitate a structured assessment. The frequency was calculated based on the number of plaques within each bin. The first bin was set to start at 0 µm, and the last bin was set at 2000 µm, ensuring full coverage of observed plaque sizes. While all bins were included in the statistical analysis, only the first 200 µm bins are shown in **Fig. 2e**, as only

a few plaques exceeded this size. LAMP1+ and IBA1+ areas were quantified using an automated GA3 recipe in NIS-Elements AR (v5.42.05) software. Images had a resolution of 512 x 512, and 1.71 µm pixel size. Briefly, maximum intensity projections of the Z-stack images were used to identify the section area, using the threshold node. Then, the area of X34+ plaques and IBA1+ area was identified with the threshold node and normalized to the section area; for X34+, minimum detectable size was set at 3 µm. The X34+ area was then expanded by 10 µm, using the circular dilate node. LAMP1+ area was measured in this peri-plaque area; LAMP1 area was then normalized by either the section area or by the peri-plaque area.

For the analysis of synapses, we focused on the cortex and took super-resolution images of X34, Synaptophysin and Homer1 using an inverted Zeiss LSM 880 microscope with an Airyscan detector in super-resolution mode. At least 4 images (containing at least 2 plaques) were taken per mouse. The system was equipped with a 63x 1.4 NA Plan-Apochromat objective lens and operated using Zen Black (version 2.3, Carl Zeiss Microscopy GmbH). Images (3x3 tiles) were taken with 1.3X electronic magnification (40 nm pixel size). The excitation lasers Argon 488, 514, He-Ne 543, 594 and 633 were used. Airyscan-acquired images were processed using the default values. Obtained images were further processed using Fiji (Imagej-win64). Briefly, a custom script was used to threshold image stacks, expand the X34 area by 5 µm and determine density and co-localization of markers. Size exclusion was set between 0.05-1.2 µm.

For imaging OPN^+ microglial cells ($IBA1^+$) around $X34^+$ and $82E1^+$ plaques, confocal fluorescent images were acquired using a Nikon AX Confocal Microscope System driven by NIS-Elements AR (v5.41.01) software, using a 40x/1.25 oil objective (Plan Apo Lambda S 40XC Sil, Nikon, #MRD73400) (2048 x 2048 resolution, 0.21 µm pixel size). Lasers were set at 405, 488, 561 and 640 nm. Resonant scanning mode was used with 16x line averaging, and minimal crosstalk was set between the channels. Thirty-one stacks were acquired using a 0.5 µm z-interval. All the quantification results were generated on six cortical images per mouse using an automated GA3 recipe in the NIS-Elements AR (v5.42.05) software. Briefly, maximum intensity projections of the Z-stack images were used to identify the area of $X34^+$ and $82E1^+$ plaques with the threshold node, with a minimum detectable size set at 3 µm for $X34^+$ plaques. These areas were then expanded by 15 and 5 µm respectively, using the circular dilate node. $IBA1^+$ and $IBA1^+OPN^+$ areas were measured in these peri-plaque areas using the threshold node. The ratio of $IBA1^+/OPN^+$ area over IBA1+ area was then normalized to the peri-plaque ($X34^+$ and $82E1^+$) areas.

Isolation of soluble and insoluble brain extracts and Aβ MSD

Soluble and insoluble brain extracts were isolated from snap-frozen brain samples. Brain weights were recorded immediately after collection. Ten volumes (w/v) P-TER buffer (Thermo Fisher Scientific, #78510) supplemented with cOmplete™ Protease Inhibitor Cocktail (Roche, # 11697498001) and PhosSTOP Phosphatase Inhibitor Cocktail (Roche, #4906845001) were added, and the tissue was homogenized in Lysing Matrix D tube (MP Biomedicals, #6913500) for 45s at 6.5 m/s. Samples were centrifuged for 5 min at 5000 g to remove debris. Subsequently, the supernatant was centrifuged for 1h at 140,000 g at 4°C in an Optima Ultracentrifuge using a TLA110 rotor to pellet the insoluble brain fraction. The supernatant (=soluble fraction) was collected and stored at -80°C and the pellet was used for guanidine

extraction. Pellets were resuspended in 2µl/mg tissue 6M GuHCl solution (GuHCl, 6M; Sigma, #G3272, 50mM Tris-HCl; Invitrogen, #AM9856; protease inhibitor cocktail, pH 7.6). Samples were sonicated with a micro-tip for 30 s at 10% amplitude, vortexed for 5 min, and incubated on a shaker for 1h at 25°C and 450 rpm. Samples were ultracentrifuged for 20 min at 227,000 g and 4°C. The supernatant, containing guanidine-soluble Aβ fractions (insoluble Aβ) were transferred into a new tube, diluted 12 times with GuHCl diluent (20mM phosphate buffer: NaH2PO4.2H2O, VWR, #928015.294, Na2HPO4.2H2O, Supelco, #106580.100; 0.4M NaCl, VWR , #27788.297; 2mM EDTA, Bioworld, #40120777-1; 10% Block Ace, Bio-Rad Laboratoris, #BUF029; 0.2% BSA, Miltenyi Biotec, #130-091-376; 0.05% NaN3, 0.075% CHAPS, Sigma-Aldrich, #C3023, protease inhibitor cocktail, pH 7.0) and stored at -80°C until use.

Aβ38, Aβ40, and Aβ42 levels in the soluble and insoluble brain extracts were quantified by Meso Scale Discovery (MSD). Standard 96-well SECTOR plates (MSD #L15XA-3) were coated with 0.5 µg ml^{-1} LTDA_38, LDA_40, or LTDA_Aβ42 capture antibodies (homemade mouse monoclonal against Aβ38, Aβ40 or Aβ42 neoepitope respectively) in PBS, pH 7.4 at 4°C overnight. Plates were washed 5 times with PBS with 0.05% Tween 20 and blocked with 0.1 % casein in PBS for 1.5 h at room temperature. Aβ standard curves were prepared with human Aβ1-38 (rPeptide #A-1078-1), Aβ1-40 (rPeptide #A-1153-1) or Aβ1-42 (rPeptide #A-1163-2). Samples were diluted according to previously determined concentrations. Diluted samples and standards were mixed 1:1 with LTDA_hAβN labelled with a sulfo-TAG detection antibody (250 ng ml^{-1}, homemade mouse monoclonal against the N-terminal sequence of human Aβ, in collaboration with Maarten Dewilde), in 0.1% casein in PBS, loaded on the blocked MSD plate, and incubated overnight at 4°C. Plates were washed 5x with PBS-T and 150µl MSD GOLD Read Buffer A (MSD #R92TG-2) was added to the wells. Plates were read with an MSD Sector Imager 2400A and MESO QuickPlex SQ 120MM (for the experiment performed on immunocompetent mice).

In vitro plaque clearance assay, image acquisition and analysis

For the *ex vivo* plaque clearance assay, microglial precursors were collected on day 25 or day 32, and differentiated into microglia-like cells using microglia differentiation medium: DMEM/F12 (Thermo Fisher Scientific, #11330032), N-acetylcysteine (5 µg ml^{-1}), insulin (500 ng ml^{-1}; Sigma-Aldrich, #I9278), Apo-Transferrin (100 µg ml^{-1}; Sigma-Aldrich, #T1147-500MG), sodium selenite (100 ng ml^{-1}; Sigma-Aldrich, #S9133), cholesterol (1.5 µg ml^{-1}; Sigma-Aldrich, #C4951) and heparan sulfate (1 µg ml^{-1}; Ams bio NL, #AMS.GAG-HS01) supplemented with 50 ng ml^{-1} interleukin-34, 50 ng ml^{-1} macrophage CSF, 10 ng ml^{-1} CX3CL1 and 25 ng ml^{-1} transforming growth factor-β, based on Abud et al[6]. Cytokines were from PeproTech. The medium was changed every other day.

In vitro plaque clearance assay was performed as previously described[7–9]. Briefly, 10-µm-thick cryosections from 6-month-old App^{NL-G-F} mouse brains[10] were collected onto poly-L-lysine-coated glass coverslips, dried at room temperature for 1 h, followed by incubation with 10 µg ml^{-1} Lecanemab, Lecanemab LALA-PG or IgG1 antibodies in PBS for 1 h at 37°C. Microglia were seeded at 5 x 10^5 cells per well in 12-well plates and incubated at 37°C with 5% CO2 for 72 h in microglia differentiation medium. For the osteopontin (OPN) stimulations, we seeded microglia onto the cryosections at 5 × 10^5 cells per well and stimulated them with human OPN

(Thermo Fisher Scientific, #120-35) at varying concentrations (0, 17, 50, 150, 450, and 1350 ng/mL). The microglia were then incubated at 37°C with 5% CO_2 for 72 hours in microglia differentiation medium. For each experiment, sections were either exposed to human microglia or left unexposed as a negative control. After incubation, coverslips were fixed with formaldehyde solution 4% for 15 minutes and washed 3 times with PBS. Afterwards, sections were permeabilized and blocked with PBS containing 0.1% Triton X-100 and 5% donkey serum at room temperature for 1h. Sections were then stained with a mouse anti-amyloid beta (N) (clone 82E1) antibody (0.2 µg ml^{-1}; IBL, #10323) overnight at 4°C to visualize Aβ plaques. The next day, sections were washed 3 times with PBS containing 0.1% Triton X (PBS-T) and incubated with donkey anti-mouse secondary antibody, Alexa Fluor™ 647 (2 µg ml^{-1}; Invitrogen, #A31571) for 1 h at room temperature, and washed 3 times with PBS-T. Next, sections were blocked with PBS-T containing 5% donkey serum at room temperature for 1 h and stained with a biotinylated mouse anti-human CD9 antibody (5 µg ml^{-1}; BioLegend, #312112) overnight at 4°C to visualize human microglia. On the next day, sections were washed 3 times with PBS-T and incubated with Streptavidin, Alexa Fluor™ 594 (2 µg ml^{-1}; Thermo Fisher Scientific, #S32356) for 1 h at room temperature, and washed 3 times with PBS-T. After washing with PBS, coverslips were placed in Mowiol mounting medium on glass slides and allowed to dry at room temperature. Large field images of the whole section were obtained using a Nikon AX Confocal Microscope System driven by NIS-Elements AR (v5.41.01) software, using 10x/0.45 air objective (Plan Apo LambdaD - WD 4.0 – Nikon, #MRD70170) with 1.6X electronic magnification (512 x 512 resolution, 2.16 µm pixel size). Tiled images were stitched together with an overlap of 15%. For excitation, 561 nm and 640 nm laser lines were used and images were acquired using the same acquisition parameters. Seven stacks were acquired using a 2 µm z-interval. Resonant scanning mode was used with 16x line averaging, and minimal crosstalk was set between the channels. Quantification of 82E1+ plaque area was performed using an automated General Analysis 3 (GA3) recipe in NIS-Elements AR (v5.42.05) software. Briefly, maximum intensity projections of the Z-stack images were used to identify the section area, using the threshold node. Then, the area of 82E1+ plaques was identified with the threshold node and normalized to the section area. All the quantification results were generated using the measurement of the whole sagittal section. For each experiment, 2-4 sections per condition were analyzed.

To confirm that $CD9^+$ cells are also GFP^+ and $IBA1^+$, we also ran an experiment using microglia derived from an H9 stem cell line stably expressing GFP under the control of a chicken β-actin promoter (CAG promoter)[11]. To visualize GFP colocalization with IBA1 and CD9, sections were stained with a mouse anti-amyloid beta (N) (clone 82E1) antibody (0.2 µg ml^{-1}; IBL, #10323) overnight at 4°C. The next day, sections were washed and incubated with donkey anti-mouse secondary antibody, Alexa Fluor™ 647 (4 µg ml^{-1}; Invitrogen, #A31571) for 1 h at room temperature, and washed 3 times with PBS-T. Next, sections were blocked with PBS-T containing 5% donkey serum at room temperature for 1h and stained with chicken anti-GFP antibody (2 µg ml^{-1}; Abcam, #ab13970) combined with biotinylated mouse anti-human CD9 antibody (5 µg ml^{-1}; BioLegend, #312112) or rabbit anti-IBA1 antibody (1 µg ml^{-1}; Wako, #019-19741) overnight at 4°C. On the next day, sections were washed 3 times with PBS-T and incubated with secondary donkey anti-chicken antibody (2 µg ml^{-1}; Jackson, #703-545-155)

and Streptavidin, Alexa Fluor™ 594 (2 µg ml^{-1}; Thermo Fisher Scientific, #S32356) or donkey anti-rabbit, Alexa Fluor™ 594 (2 µg ml^{-1}; Invitrogen, #A21207) for 1 h at room temperature, and washed 3 times with PBS-T. After washing with PBS, coverslips were placed in Mowiol mounting medium on glass slides and allowed to dry at room temperature.

Isolation of human microglia

Human microglia isolation from the mouse brain was performed as previously described[12]. After perfusion with ice-cold heparinized PBS, one hemisphere (without the cerebellum and olfactory bulb) was placed in FACS buffer (PBS containing 2% FCS and 2 mM EDTA) supplemented with 5 µM actinomycin D (ActD; Sigma-Aldrich, # A1410-5MG) for transcriptomics. Brains were mechanically and enzymatically dissociated using Miltenyi Neural Tissue Dissociation Kit P (Miltenyi, #130-092-628) supplemented with 5 µM ActD. Next, samples were passed through a 70-µm strainer (BD2 Falcon), washed in 10 ml of ice-cold FACS buffer with 5 µM ActD and spun at 300 g for 15 min at 4 °C. ActD was kept during collection and enzymatic dissociation of the tissue to prevent artificial activation of human microglia during the procedure[13]. ActD was removed from the myelin removal step to prevent toxicity derived from long-term exposure. Following dissociation, myelin was removed by resuspending pelleted cells in 30% isotonic Percoll (GE Healthcare, #17-5445-02) and centrifuging at 300 g for 15 min at 4 °C. Accumulating layers of myelin and cellular debris were discarded and Fc receptors were blocked in FcR blocking reagents (mouse, 0.1 mg ml^{-1}, Miltenyi, #130-092-575; human, 0.1 mg ml^{-1}, Miltenyi, # 130-059-901) in cold FACS buffer for 10 min at 4 °C. Next, cells were washed in 5 ml of FACS buffer and pelleted cells were incubated with the following antibodies: PE-Pan-CD11b (20 µg ml^{-1}; Miltenyi, #130-113-806), BV421-mCD45 (2 µg ml^{-1}; BD Biosciences, #563890), APC-hCD45 (20 µg ml^{-1}; BD Biosciences, #555485), Total-Seq A cell hashing antibodies (2 µg ml^{-1}; BioLegend) and viability dye (0.5 µg ml^{-1}; eFluor 780, Thermo Fisher Scientific, #65-0865-14), in cold FACS buffer during 30 min at 4 °C. After incubation, cells were washed, and the pellet was resuspended in 400 µl of FACS buffer and passed through a 35-µm strainer before sorting. For sorting, the cell suspension was loaded into the input chamber of a MACSQuant Tyto Cartridge, and human cells were sorted based on CD11b and hCD45 expression at 4 °C (MACSQuantify™ Tyto®).

***In vivo* phagocytosis assay**

Starting from 6 months of age, *App*$^{NL\text{-}G\text{-}F}$ mice xenotransplanted with human microglia were treated with Lecanemab or IgG1 for 8 weeks. On the day of the experiment, they were injected i.p. with 10mg/kg Methoxy-X04 (Tocris Biosciences, #4920) (protocol adapted from (Lau et al. 2021)) reconstituted in Kolliphor-EL (Sigma-Aldrich, #C5125). Three hours later, mice were euthanized, and microglia was isolated as described above. Cells were washed in 5 ml of FACS buffer and pelleted cells were incubated with the following antibodies: PE-Pan-CD11b (20 µg ml^{-1}, Miltenyi, #130-113-806), AF488-mCD45 (2 µg ml^{-1}, Biolegend, #109815), APC-hCD45 (2 µg ml^{-1}, BD Biosciences, #555485), and viability dye (0.5 µg ml^{-1}, eFluor 780, Thermo Fisher Scientific, #65-0865-14), in cold FACS buffer for 30 min at 4 °C. After incubation, cells were washed and fixed with eBioscience Foxp3 Fixation/Permeabilization kit (eBioscience, #00-5521-00) for 30 min at 4 °C. For intracellular CD68 staining, fixed cells were permeabilized (eBioscience #00-8333-56), washed and incubated in PE Vio615 CD68 (10 µg ml^{-1}, Miltenyi,

#130-114-656) in permeabilization buffer overnight at 4 °C. Cells were washed with FACS buffer, the pellet was resuspended in 500 µl of FACS buffer and passed through a 35-µm strainer prior to FACS acquisition. Flow data was acquired on a BD Fortessa (BD FACSDiva Software, version 9.7). Dead cells and doublets were gated out prior to downstream analysis. Human microglia were identified by gating on hCD45. Methoxy-X04 gating was set with negative controls (xenografted *App*$^{Hu/Hu}$ mice injected with Methoxy-X04). Methoxy-X04 populations within the hCD45 and within the CD68-positive gate were analysed for Methoxy-X04 incorporation. FACS data was analysed with the FlowJo software (v10.8.1).

Single-cell sequencing data analysis

Sample demultiplexing across single cell libraries

Demultiplexing of cells to their sample of origin was performed as described in Stoeckius *et al.*[14]. In brief, cell barcodes were filtered to include only those detected in both RNA and HTO data. The HTO assays were normalized using the centered log-ratio (CLR) transformation. Seurat's *HTODemux()* function was run with default parameters on the hashtag-count matrices to demultiplex the cells. Cells that could not be assigned to an HTO and cells that were positive for more than one HTO were deemed as negatives and inter-sample doublets, respectively, and removed from further analysis.

Microglial cell state assignment

Cell state identities were assigned to each of the clusters based on the expression of marker genes, as determined in "Single-cell differential gene expression analysis" and visualized by Seurat's *DotPlot()* (**Extended Data Fig. 4f**).

Gene Set Enrichment Analyses

Gene Set Enrichment Analyses (GSEA)[15] was performed using the clusterProfiler[16] package (v4.6.2). The outputs of differential expression analyses between conditions (in the scRNA-seq analyses) or in function of distance to pathology (in the Nova-ST data) were ranked according to each gene's log2(FoldChange). ClusterProfiler's *gseKEGG()* and *GSEA()* functions were run on this ranked list to calculate gene set enrichment scores for KEGG pathways (**Fig. 1f-g**, **Extended Data Fig. 2d-e**) and WGCNA modules (**Fig. 4d**, **Fig. 5b**, **Extended Data Fig. 5e**), while *gsePathway()* from the ReactomePA[17] (v1.42.0, R) package was run to do the same for Reactome pathways (**Extended Data Fig. 4e**). The following parameters were used: *nPerm* = 1000, *minGSSize* = 40, *maxGSSize* = 800, *pvalueCutoff* = 0.05, qvalueCutoff = 0.05, *pAdjustMethod* = "BH". The GSEA enrichment profiles of selected signatures were plotted using enrichr's gseaplot2() function.

Weighted Gene Co-expression Network Analysis (WGCNA)

To perform co-expression network analysis with WGCNA[18], we employed High-Definition Weighted Gene Co-expression Network Analysis (hdWGCNA)[19] (v0.3.0, R), specifically designed for analysis of high dimensional snRNA-seq data. Briefly, this approach groups transcriptionally similar cells into "metacells", so as to overcome the issue of sparsity of single cell data and thus providing more robust gene-gene correlation estimates. First, we filtered out genes expressed in less than 5% of all cells. We next generated metacells per sample with the *MetacellsByGroups()* function which employs a k-Nearest Neighbors (KNN) algorithm, using the parameters *target_metacells*=200, *group.by*="sample",

reduction="pca", *k*=25, *max.shared*=10. Next, we used the *TestSoftPowers()* function to determine the optimal soft power threshold for constructing a "signed hybrid" co-expression network. We set the minimum soft power threshold at the minimum value with a Scale Free Topology Model Fit of at least 0.9, so as to retain robust gene-gene correlations while eliminating weak links in the adjacency matrix. Finally, we performed a signed-hybrid network construction and module detection with the *ConstructNetwork()* function. The module dendrogam was visualized using the *PlotDendrogram*() function (**Extended Data Fig. 5a**). The *ModuleEigengenes()* function was run to determine the module eigengenes (MEs), or the first principal components of the expression matrix specific to each of the modules. The intra-modular connectivity (kME) of each gene, representing the correlation of the gene with its ME, was calculated using *SignedKME*(). The 10 genes with the highest kMEs per module were identified as hub genes and their connectivities were plotted using the ggraph and tidygraph packages (v2.1.0 and v1.2.3, respectively, R) (**Fig. 4e**).

Gene Set Enrichment Visualization

To calculate and visualize each cell's enrichment of gene sets of interest (such as markers for microglial states and WGCNA modules), we used Seurat's *AddModuleScore()* function. In brief, this function calculates the activity of a gene set in each cell by comparing the mean abundance level of the genes of interest against the average abundance of sets of random control genes which have a similar average expression level. The calculated scores were visualized on the UMAP (**Extended Data Fig. 5c**).

Gene Set Overrepresentation Analyses

Over-representation of Gene Ontology[20,21] terms (Biological Processes and Molecular Functions) in the WGCNA modules was assessed with clusterProfiler's *enrichGO()* function (**Fig. 4e**, **Supplementary Table 2**). The analyses were run using the parameters: *minGSSize* = 10, *maxGSSize* = 500, *pvalueCutoff* = 0.05, *qvalueCutoff* = 0.2, *pAdjustMethod* = "BH".

References

1. Poovathingal, S., Davie, K. & Aerts, S. Nova-ST Spatial Transcriptomics protocol v1. Preprint at https://doi.org/10.17504/protocols.io.3byl4925jgo5/v1 (2024).
2. Poovathingal, S. *et al.* Nova-ST: Nano-patterned ultra-dense platform for spatial transcriptomics. *Cell Reports Methods* **4**, 100831 (2024).
3. Poovathingal, S., Davie, K. & Aerts, S. Nova-ST Chip Preparation Protocol v1. Preprint at https://doi.org/10.17504/protocols.io.n92ld835ov5b/v1 (2024).
4. Franklin, K. B. J. & Paxinos, G. *Paxino's and Franklin's the Mouse Brain in Stereotaxic Coordinates: Compact 5th Edition.* (Elsevier Science & Technology, San Diego, 2019).
5. Bankhead, P. *et al.* QuPath: Open source software for digital pathology image analysis. *Sci Rep* **7**, 16878 (2017).
6. Abud, E. M. *et al.* iPSC-Derived Human Microglia-like Cells to Study Neurological Diseases. *Neuron* **94**, 278-293.e9 (2017).
7. Bard, F. *et al.* Peripherally administered antibodies against amyloid beta-peptide enter the central nervous system and reduce pathology in a mouse model of Alzheimer disease. *Nat Med* **6**, 916–919 (2000).

8. Claes, C. *et al.* Human stem cell–derived monocytes and microglia-like cells reveal impaired amyloid plaque clearance upon heterozygous or homozygous loss of TREM2. *Alzheimer’s & Dementia* **15**, 453–464 (2019).
9. Xiang, X. *et al.* TREM2 deficiency reduces the efficacy of immunotherapeutic amyloid clearance. *EMBO Mol Med* **8**, 992–1004 (2016).
10. Saito, T. *et al.* Single App knock-in mouse models of Alzheimer’s disease. *Nat Neurosci* **17**, 661–663 (2014).
11. Balusu, S. *et al. Long Noncoding RNA MEG3 Activates Neuronal Necroptosis in Alzheimer’s Disease*. http://biorxiv.org/lookup/doi/10.1101/2022.02.18.480849 (2022) doi:10.1101/2022.02.18.480849.
12. Mancuso, R. *et al.* Xenografted human microglia display diverse transcriptomic states in response to Alzheimer’s disease-related amyloid-β pathology. *Nat Neurosci* (2024) doi:10.1038/s41593-024-01600-y.
13. Marsh, S. E. *et al.* Dissection of artifactual and confounding glial signatures by single-cell sequencing of mouse and human brain. *Nat Neurosci* **25**, 306–316 (2022).
14. Stoeckius, M. *et al.* Cell Hashing with barcoded antibodies enables multiplexing and doublet detection for single cell genomics. *Genome Biol* **19**, 224 (2018).
15. Subramanian, A. *et al.* Gene set enrichment analysis: A knowledge-based approach for interpreting genome-wide expression profiles. *Proc. Natl. Acad. Sci. U.S.A.* **102**, 15545–15550 (2005).
16. Wu, T. *et al.* clusterProfiler 4.0: A universal enrichment tool for interpreting omics data. *The Innovation* **2**, 100141 (2021).
17. Yu, G. & He, Q.-Y. ReactomePA: an R/Bioconductor package for reactome pathway analysis and visualization. *Mol. BioSyst.* **12**, 477–479 (2016).
18. Langfelder, P. & Horvath, S. WGCNA: an R package for weighted correlation network analysis. *BMC Bioinformatics* **9**, 559 (2008).
19. Morabito, S., Reese, F., Rahimzadeh, N., Miyoshi, E. & Swarup, V. hdWGCNA identifies co-expression networks in high-dimensional transcriptomics data. *Cell Reports Methods* **3**, 100498 (2023).
20. Ashburner, M. *et al.* Gene Ontology: tool for the unification of biology. *Nat Genet* **25**, 25–29 (2000).
21. The Gene Ontology Consortium *et al.* The Gene Ontology knowledgebase in 2023. *GENETICS* **224**, iyad031 (2023).
