# Supplementary material for: The Alzheimer’s therapeutic Lecanemab attenuates Aβ pathology by inducing an amyloid-clearing program in microglia
Source: Nat Neurosci. 2025 Nov 24;29(1):100–10. doi: 10.1038/s41593-025-02125-8 (PMC12779577; doi:10.1038/s41593-025-02125-8)
Supplement: Supplementary file 2 — Reporting Summary [file 41593_2025_2125_MOESM2_ESM.pdf]

nature portfolio

Corresponding author(s): Bart De Strooper, Giulia Albertini

Last updated by author(s): Sep 12, 2025

Reporting Summary

Nature Portfolio wishes to improve the reproducibility of the work that we publish. This form provides structure for consistency and transparency in reporting. For further information on Nature Portfolio policies, see our Editorial Policies and the Editorial Policy Checklist.

Statistics

For all statistical analyses, confirm that the following items are present in the figure legend, table legend, main text, or Methods section.

n/a	Confirmed	
☐	☒	The exact sample size (*n*) for each experimental group/condition, given as a discrete number and unit of measurement
☐	☒	A statement on whether measurements were taken from distinct samples or whether the same sample was measured repeatedly
☐	☒	The statistical test(s) used AND whether they are one- or two-sided<br>*Only common tests should be described solely by name; describe more complex techniques in the Methods section.*
☐	☒	A description of all covariates tested
☐	☒	A description of any assumptions or corrections, such as tests of normality and adjustment for multiple comparisons
☐	☒	A full description of the statistical parameters including central tendency (e.g. means) or other basic estimates (e.g. regression coefficient) AND variation (e.g. standard deviation) or associated estimates of uncertainty (e.g. confidence intervals)
☐	☒	For null hypothesis testing, the test statistic (e.g. *F*, *t*, *r*) with confidence intervals, effect sizes, degrees of freedom and *P* value noted<br>*Give P values as exact values whenever suitable.*
☒	☐	For Bayesian analysis, information on the choice of priors and Markov chain Monte Carlo settings
☒	☐	For hierarchical and complex designs, identification of the appropriate level for tests and full reporting of outcomes
☒	☐	Estimates of effect sizes (e.g. Cohen's *d*, Pearson's *r*), indicating how they were calculated

Our web collection on statistics for biologists contains articles on many of the points above.

Software and code

Policy information about availability of computer code

Data collection	The raw files for single cell RNA-seq analysis were demultiplexed and aligned by Cellranger (v.6.1.2) against the human/mouse reference genome (GRCh38 and mm10). Nova-ST data was pre-processed, mapped to the joint human/mouse reference genome and quantified using the NovaScope Pipeline (v1.111), spatula (v1.0.0) and STAR (v2.7.11a). Mapped and quantified data was loaded and binned into hexagonal bins (TDs) with a width, and center-to-center distance of 40µm using custom Python code. Microscopy data was collected using Nikon AX Confocal Microscope System driven by NIS-Elements AR (5.41.01), Nikon NiE 8-staged Microscope driven by NIS-Elements AR (6.10.01) software and inverted Zeiss LSM 880 microscope with Airyscan detector operated using Zen Black (version 2.3). MSD data was aquired using MSD Discovery Workbench v4. Flow cytometry data was collected using MACSQuantify™ Tyto® Software 1.0 and BD FACSDiva Software (version 9.7).
Data analysis	Raw scRNA-seq count matrices were imported in R (v4.2.3) for data analysis. Single cell datasets were analyzed using the Seurat R package pipeline (v.4.1.1) Nova-ST expression matrices were read into python processing. TD expression profiles were pre-processed and analyzed using the Scanpy package (v1.9.8) in python as well as the edgeR (v3.40.0) and Seurat (v4.1.1) R packages. Nova-ST fluorescence images were aligned to spatial data using the Landmark Correspondences plugin in Fiji. All other packages and their versions used for the analyses of this study are reported in the Methods Section. These include: geopandas (v0.12.0) , Scipy (v1.10.1), squidpy (v1.2.3), DoubletFinder (v2.0.3), MASC (v0.1.0), ggplot2 (v3.5.2), clusterProfiler (v4.6.2), hdWGCNA (v0.3.0), ReactomePA (v1.42.0), ggraph (v2.1.0), tidygraph (v1.2.3). Single cell and Nova-ST analyses will be made available at: https://github.com/mmzielonka/AlbertiniZielonka_Lecanemab_2025.git. For specific statistical tests and visualizations, we also used GraphPad Prism (v9.0). Microscopy data was analyzed using Qupath (v0.4.3), NIS-Elements AR (v5.42.05) and Fiji (ImageJ-win64) softwares. Flow cytometry data was analyzed with FlowJo software (v10.8.1).

For manuscripts utilizing custom algorithms or software that are central to the research but not yet described in published literature, software must be made available to editors and reviewers. We strongly encourage code deposition in a community repository (e.g. GitHub). See the Nature Portfolio guidelines for submitting code & software for further information.

Data

Policy information about availability of data

All manuscripts must include a data availability statement. This statement should provide the following information, where applicable:

- Accession codes, unique identifiers, or web links for publicly available datasets
- A description of any restrictions on data availability
- For clinical datasets or third party data, please ensure that the statement adheres to our policy

Transcriptomic data generated in this study are available at Gene Expression Omnibus (GEO) database under accession numbers GSE297667 (spatial transcriptomics data) and GSE297665 (scRNA-seq data). Other data are available upon request.The source code used in this study is available via GitHub at https://github.com/mmzielonka/AlbertiniZielonka_Lecanemab_2025.git and Zenodo at https://doi.org/10.5281/zenodo.17098381

Research involving human participants, their data, or biological material

Policy information about studies with human participants or human data. See also policy information about sex, gender (identity/presentation), and sexual orientation and race, ethnicity and racism.

Reporting on sex and gender	No human participants were used in this study
Reporting on race, ethnicity, or other socially relevant groupings	No human participants were used in this study
Population characteristics	No human participants were used in this study
Recruitment	No human participants were used in this study
Ethics oversight	No human participants were used in this study

Note that full information on the approval of the study protocol must also be provided in the manuscript.

Field-specific reporting

Please select the one below that is the best fit for your research. If you are not sure, read the appropriate sections before making your selection.

☒ Life sciences ☐ Behavioural & social sciences ☐ Ecological, evolutionary & environmental sciences

For a reference copy of the document with all sections, see nature.com/documents/nr-reporting-summary-flat.pdf

Life sciences study design

All studies must disclose on these points even when the disclosure is negative.

Sample size	For single cell RNA-seq analysis, sample size was estimated based on previous experiments performed in the lab (Mancuso, Fattorelli, Martinez-Muriana et al., 2024). To calculate the number of mice needed for IF and MSD ELISA, we conducted a priori power analyses using G*Power (sample size noted throughout the test). The expected effect size was based on previous publications or, when possible, data previously generated in the lab. Alpha level and power were set at 5% and 80%, respectively, and the statistical model used for the sample size calculation was a t-test.
Data exclusions	We excluded from the single cell RNA sequencing dataset 1 mouse that showed extremely low cell numbers (probably due to technical errors). For all the ELISA data, statistical outliers (caused by technical errors) were identified using the ROUT test in Prism10 (Q=1%) and excluded from further analysis. No additional mice were excluded from the study.
Replication	For scRNA-seq analysis the final high-quality microglia dataset consisted of 22,420 cells from 12 independent mice and 5 sequencing libraries. For all statistical test and figures, each data point represents the single cell data set of one mouse.<br>For Nova-ST analysis, the dataset consisted of spatial expression profiles from 4 sagittal sections, resulting in the analysis of 32,568 cortical tissue domains (hexbins with width and center-to-center distance of 40um) passing a pre-defined human count threshold.<br>All n values represent individual animals, unless stated otherwise (i.e., ex vivo plaque clearance assay, where each n represent an independent experiment). Mice come from 4 independent experiments (independent microglial cells differentiations, independent antibodies production).<br>All attempts at replication were successful.
Randomization	Animals were randomly assigned to conditions and conditions were randomized to account for potential ordering effects. To avoid litter bias in the mouse experiments, experimental groups were composed of animals from different litters randomly distributed.
Blinding	For all experiments, analyses were conducted blindly to the experimental condition.

Reporting for specific materials, systems and methods

We require information from authors about some types of materials, experimental systems and methods used in many studies. Here, indicate whether each material, system or method listed is relevant to your study. If you are not sure if a list item applies to your research, read the appropriate section before selecting a response.

Materials & experimental systems

n/a	Involved in the study
☐	☒ Antibodies
☐	☒ Eukaryotic cell lines
☒	☐ Palaeontology and archaeology
☐	☒ Animals and other organisms
☒	☐ Clinical data
☒	☐ Dual use research of concern
☒	☐ Plants

Methods

n/a	Involved in the study
☒	☐ ChIP-seq
☐	☒ Flow cytometry
☒	☐ MRI-based neuroimaging

Antibodies

Antibodies used

Lecanemab (10 mg/kg in vivo, 10 ug/mL in vitro; home-made and Genscript), Lecanemab LALA-PG (10 mg/kg in vivo, 10 ug/mL in vitro; Genscript), IgG1 isotype control (10 mg/kg in vivo, 10 ug/mL in vitro; Imtec Diagnostics, #LT9005), mAb158 (10 mg/kg; Genscript), mAb158 LALA-PG (10 mg/kg; Genscript) and IgG2a isotype control (10 mg/kg; Leinco, #P381)
Mouse anti-Human Amyloidβ (N) (82E1) (0.2ug/ml, IBL, #10323)
Rabbit anti-human P2RY12 (0.2 µg/ml; Atlas Antibodies, #HPA013796)
Guinea-pig anti-IBA1 antibody (2 µg/ml, Synaptic System, #234 308)
Rat anti-LAMP1 antibody (4 µg/ml; DSHB, #1D4B-c)
Rabbit anti-IBA1 antibody (1 µg/ml−1; Wako, #019-19741)
Goat anti-osteopontin/OPN antibody (2 µg/ml; R&D System, AF808)
PE-CD11b (20µg/ml, Miltenyi, 130-113-806)
APC-hCD45 (20µg/ml, BD Biosciences, #555485)
AF488-mCD45 (2 µg/ml, Biolegend #109815)
BV421-mCD45 (2µg/ml, BD Biosciences, #563890)
PE Vio615 CD68 (10 µg/ml, Miltenyi, #130-114-656)
TotalSeqTM-A cell hashing antibodies (2µg/ml, Biolegend)
LTDA_Aβ42, LTDA_Aβ40 and LTDA_Aβ38 (0.5µg/ml, home-made)
LTDA_hAβN (250ng/ml, home-made)
goat anti-Human IgG- Alexa Fluor™ 647 (80 µg/mL, ThermoFisher Scientific, #A-21445)
rabbit anti-β-Amyloid (D54D2)- Alexa Fluor™ 594 (80 µg/mL, Bioké, #35363S)
mouse anti-Human CD45- Alexa Fluor™ 488 (80 µg/mL, BioLegend, #304017)
rabbit anti-Homer 1 antibody (2 µg/ml; Synaptic systems, #160003)
mouse anti-Synaptophysin antibody (2 µg/ml; Synaptic systems, #101011)
mouse anti-human HLA antibody (5 µg/ml; Abcam, ab7856)
Biotin mouse anti-human CD9 (5-10ug/ml, Biolegend, #312112)
rabbit anti-human CD68 (4 µg/ml; Abcam, #ab213363)
chicken anti-GFP antibody (2 µg/ml; Abcam, #ab13970)

Validation

Lecanemab, Lecanemab LALA-PG, IgG1 isotype control, mAb158, mAb158 LALA-PG and IgG2a isotype control: the purity of the antibodies was estimated to be above 75% by densitometric analysis of the Coomassie Blue-stained SDS-PAGE gel under non-reducing conditions. Binding to Aβ1-42 (rPeptide, #A-1163-2), was confirmed by ELISA and Dot Blot. Also, the human antibodies have been used and validated in an additional publication (Fertan E, Lam JYL, et al. Single-molecule characterisation of soluble beta-amyloid aggregate binding by Aducanumab, Lecanemab, Gantenerumab, and Donanemab. In press in Alzheimer's & Dementia (Bioarchive: doi 10.11.617910)).
Mouse anti-Human Amyloidβ (N) (82E1): validated in Horikoshi Y, et al. Development of Abeta terminal end-specific antibodies and sensitive ELISA for Abeta variant. Biochem Biophys Res Commun. 2004 Jul 2;319(3):733-7.
Rabbit anti-human P2RY12: Validated by the company by IHC on sections of human cerebral cortex and liver tissue.
Guinea pig anti-IBA1 antibody: Validated by the company by IHC on rat, mouse and human brain sections.
Rat anti-LAMP1 antibody: Validated by IHC by McNiven Laboratory, Mayo Clinic, Rochester, on AML12 mouse hepatocyte cells. Hughes EN, August JT. Characterization of plasma membrane proteins identified by monoclonal antibodies. J Biol Chem. 1981 Jan 25;256(2):664-71.
Rabbit anti-IBA1 antibody: Validated by the company by IHC on mouse brain sections.
Goat anti-osteopontin/OPN antibody: Validated by the company by IHC on mouse brain, spleen and thymus sections. Used on human brain sections in: Lawrence AR, et al. Microglia maintain structural integrity during fetal brain morphogenesis. Cell. 2024 Feb 15;187(4):962-980.e19.
PE-CD11b: validated in splenocytes from BALB/c mice were stained with CD11b antibodies or with the corresponding REA control antibodies.
APC-hCD45: validated on human peripheral blood lymphocytes, stained with either APC Mouse IgG1, or κ isotype control.
AF488-mCD45: validated by the company on mouse splenocytes.
BV421-mCD45: validated on mouse splenic leucocytes preincubated with Purified Rat Anti-Mouse CD16/CD32 antibody and then stained with either BD Horizon™ BV421 Rat IgG2b or κ Isotype Control.

LTDA_Aβ42, LTDA_Aβ40, LTDA_Aβ38 and LTDA_hAβN were generated in-house. LTDA_Aβ42, LTDA_Aβ40 and LTDA_Aβ38 are recombinant mouse monoclonal antibody that recognize the carboxy terminus of Aβ42, Aβ40 and Aβ38 (do not recognize other Aβs, ELISA). They react equally potent to rodent Aβ42, 40 and 38.LTDA_hAβN is a recombinant mouse monoclonal antibody with epitope at first 7 residues of human Aβ; requires β-cleavage of APP to recognize human APP-β-CTF and Aβ 1-X. Tihs antibody was tested for human Aβ (western blot and ELISA) and β-CTF(western blot), control rodent Aβ and β-CTF are not recognized.
goat anti-Human IgG- Alexa Fluor™ 647: validated by WB, ICC, IF. Antibody specificity was demonstrated by detection of differential basal expression of IgG across cell lines owing to their inherent genetic constitution.
rabbit anti-β-Amyloid (D54D2)- Alexa Fluor™ 594: This antibody recognizes endogenous levels of total β-amyloid peptide (Aβ). The antibody detects several isoforms of Aβ, such as Aβ-37, Aβ-38, Aβ-39, Aβ-40, and Aβ-42. Validated by the company by WB and IF.
mouse anti-Human CD45- Alexa Fluor™ 488: validated by the company on human peripheral blood lymphocytes.
rabbit anti-Homer 1 antibody: validated by the company on WB, ICC, IHC. Cross-reactivity of the serum to Homer 2 and 3 was removed by pre-adsorption with Homer 2 (aa 1 - 176) and Homer 3 (aa 1 - 177).
mouse anti-Synaptophysin antibody: validated by the company on WB, ICC, IHC. Validated on KO (PubMed, 31940485).
mouse anti-human HLA antibody: tested by the company by IHC-P and WB on human tissues.
Biotin mouse anti-human CD9:Validated by the company by flow cytometry on human platelets.
rabbit anti-human CD68: Specificity and sensitivity confirmed in IHC with multi-tissue microarray (TMA) validation.
chicken anti-GFP antibody: validated by the company for use in ICC/IF and WB.

Eukaryotic cell lines

Policy information about cell lines and Sex and Gender in Research

Cell line source(s)	We used human embryonic stem cells, WA09 (H9), female, obtained from WiCell Research Institute (catalog #WA09), RRID: CVCL_9773.
Authentication	Cell lines were authenticated by the providers by Karyotyping and whole genome sequencing, and have been tested for pluripotency (https://hpscreg.eu/cell-line/WAe009-A)
Mycoplasma contamination	All the lines used were regularly tested negative for mycoplams
Commonly misidentified lines (See ICLAC register)	None of the cell lines used in this study is known to be cross-contaminated or otherwise misidentified, and is not listed in the Register of Misidentified Cell Lines from ICLAC

Animals and other research organisms

Policy information about studies involving animals; ARRIVE guidelines recommended for reporting animal research, and Sex and Gender in Research

Laboratory animals	Species: Mus Musculus<br>Strains used: App^NL-G-F (Apptm3.1Tcs, C57BL/6 background; RIKEN, Takaomi Saido; RRID: IMSR_RBRC06344); App^NL-G-F Csf1r^ΔFIRE/ΔFIRE (mixed background, generated in-house at KU Leuven); immunocompromized App^NL-G-F (Rag2^tm1.1Flv; Csf1^tm1(CSF1)Flv; Il2rg^tm1.1Flv; Apptm3.1Tcs; mixed background, generated in-house at KU Leuven); App^Hu (App^em1Bdes, C57BL/6 background; mixed background, generated in-house at KU Leuven); CD1 foster mothers (Charles River Laboratories, strain code 022). Mice were used from P4 (grafting) to 6-8 months of age or, if grafting was not involved, from 4 to 6 months of age.<br>All mice were housed in a specific pathogen-free facility under a 14 h light/10 h dark cycle, at an ambient temperature of 21 °C and 40-60% humidity, in groups of two to five animals, with food and water provided ad libitum.
Wild animals	No wild animals were used
Reporting on sex	Experimental groups were balanced in terms of the sex of the mice
Field-collected samples	No field-collected samples were used in this study
Ethics oversight	Animal experiments were approved by the local Ethical Committee of Laboratory Animals of the KU Leuven (government licence LA1210579, ECD project numbers P125/2022 and P132/2022) following local and EU guidelines.

Note that full information on the approval of the study protocol must also be provided in the manuscript.

Plants

Seed stocks	No plants were used in this study
Novel plant genotypes	No plants were used in this study
Authentication	No plants were used in this study

Flow Cytometry

Plots

Confirm that:

☒ The axis labels state the marker and fluorochrome used (e.g. CD4-FITC).

☒ The axis scales are clearly visible. Include numbers along axes only for bottom left plot of group (a 'group' is an analysis of identical markers).

☒ All plots are contour plots with outliers or pseudocolor plots.

☒ A numerical value for number of cells or percentage (with statistics) is provided.

Methodology

Sample preparation	For purification of microglia for downstream single cell RNA sequencing analysis, we used the following protocol. After perfusion with ice-cold heparinized PBS, one hemisphere (without cerebellum and olfactory bulb) was placed in FACS buffer (PBS containing 2% FCS and 2 mM EDTA) supplemented with 5 µM actinomycin D (ActD; Sigma, # A1410-5MG) for transcriptomics. Brains were mechanically and enzymatically dissociated using Miltenyi Neural Tissue Dissociation Kit P (Miltenyi, #130-092-628) supplemented with 5 µM ActD. Next, samples were passed through a 70-µm strainer (BD2 Falcon), washed in 10 ml of ice-cold FACS buffer with 5 µM ActD and spun at 300 g for 15 min at 4 °C. ActD was kept during collection and enzymatic dissociation of the tissue to prevent artificial activation of human microglia during the procedure12. ActD was removed from the myelin removal step to prevent toxicity derived from long-term exposure. Following dissociation, myelin was removed by resuspending pelleted cells in 30% isotonic Percoll (GE Healthcare, #17-5445-02) and centrifuging at 300 g for 15 min at 4 °C. Accumulating layers of myelin and cellular debris were discarded and Fc receptors were blocked in FcR blocking reagents (mouse, 1:10, Miltenyi, #130-092-575; human, 1:10, Miltenyi, # 130-059-901) in cold FACS buffer for 10 min at 4 °C. Next, cells were washed in 5 ml of FACS buffer and pelleted cells were incubated with the following antibodies: PE-Pan-CD11b (1:50, Miltenyi, #130-113-806), BV421-mCD45 (1:500, BD Biosciences, #563890), APC-hCD45 (1:50, BD Biosciences, #555485), Total-Seq A cell hashing antibodies (1:500, BioLegend) and viability dye (1:2,000, eFluor 780, Thermo Fisher Scientific, #65-0865-14), in cold FACS buffer during 30 min at 4 °C. After incubation, cells were washed, and the pellet was resuspended in 400 µl of FACS buffer and passed through a 35-µm strainer before sorting. For sorting, the cell suspension was loaded into the input chamber of a MACSQuant Tyto Cartridge, and human cells were sorted based on CD11b and hCD45 expression at 4 °C.<br>For in vivo phagocytosis assay, microglia were isolated as described above. Cells were washed in 5 ml of FACS buffer and pelleted cells were incubated with the following antibodies: PE-Pan-CD11b (20 µg ml−1, Miltenyi, #130-113-806), AF488-mCD45 (2 µg ml−1, Biolegend #109815), APC-hCD45 (2 µg ml−1, BD Biosciences, #555485), and viability dye (0.5 µg ml−1, eFluor 780, Thermo Fisher Scientific, #65-0865-14), in cold FACS buffer for 30 min at 4 °C. After incubation, cells were washed and fixed with eBioscience Foxp3 Fixation/Permeabilization kit (eBioscience, #00-5521-00) for 30 min at 4 °C. For intracellular CD68 staining, fixed cells were permeabilized (eBioscience, #00-8333-56), washed and incubated in PE Vio615 CD68 (10 µg ml −1, Miltenyi, #130-114-656) in permeabilization buffer overnight at 4 °C. Cells were washed with FACS buffer, the pellet was resuspended in 500 µl of FACS buffer and passed through a 35-µm strainer prior to FACS acquisition. Flow data was acquired on a BD Fortessa (BD FACSDiva Software, version 9.7). FACS data was analysed with FlowJo software (v10.8.1).
Instrument	MACSQuant Tyto, BD Fortessa .
Software	MACSQuantify™ Tyto® Software 1.0, BD Fortessa (BD FACSDiva Software, version 9.7) and FlowJo software (v10.8.1).
Cell population abundance	Purity was assessed by flow cytometry. As shown in Extended Data Fig. 3, within the myeloid populations (CD11b+), the mouse host (mCD45+) and human transplanted microglia (hCD45+) form two clearly distinct populations. The population of interest is the human transplanted microglia (CD11b+ hCD45+). The percentage of human microglia within the CD11b+ cells ranged from 18% to 80%, with an average of 49.6%. Within this population, we calculated the percentage of cells positive for Methoxy-x04, as well as the Methoxy-x04+ cells within the CD68-high gate.
Gating strategy	BSC and SSC were used to filter debri and doublet discrimination. e780 (Thermo Fisher) was used as a viability marker. All analyses were performed on viable singlets. Dead cells and doublets were gated out prior to downstream analysis. Human microglia were identified by gating on hCD45. Methoxy-x04 gating was set with negative controls (xenografted AppHu mice injected with Methoxy-x04). Methoxy-x04 populations within the hCD45 and within the CD68-positive gate were analysed for Methoxy-X04 incorporation.

☒ Tick this box to confirm that a figure exemplifying the gating strategy is provided in the Supplementary Information.
